# Comprehensive pan-cancer analysis reveals NUSAP1 is a novel predictive biomarker for prognosis and immunotherapy response

**DOI:** 10.7150/ijbs.80017

**Published:** 2023-09-04

**Authors:** Hong Zheng, Minghao Wang, Shiyu Zhang, Dongxue Hu, Qiaoyun Yang, Ming Chen, Xia Zhang, Yi Zhang, Jigang Dai, Yih-Cherng Liou

**Affiliations:** 1 Department of Biological Sciences, Faculty of Science, National University of Singapore, Singapore, Singapore.; 2 Department of Thoracic Surgery, Xinqiao Hospital, Army Medical University, Chongqing, China.; 3 Department of Breast and Thyroid Surgery, Southwest Hospital, Army Medical University, Chongqing, China.; 4 Department of Medical Oncology, The First Affiliated Hospital, Zhejiang University School of Medicine, Hangzhou, China.; 5 Institute of Pathology and Southwest Cancer Center, Southwest Hospital, Army Medical University and Key Laboratory of Tumor Immunopathology, Ministry of Education of China, Chongqing, China.

**Keywords:** NUSAP1, pan-cancer, biomarker, prognosis, immunotherapy.

## Abstract

Nucleolar and spindle-associated protein 1 (NUSAP1) is a microtubule-associated protein that plays a crucial role in mitosis. Despite initial reports suggesting a potential involvement of NUSAP1 in tumor progression and malignant cell regulation, there has been no systematic analysis of its role in the tumor immune microenvironment, nor its predictive value for prognosis and immunotherapy response across different cancer types. In this study, we analyze NUSAP1 mRNA and protein expression levels in various human normal and tumor tissues, using data from TCGA, GTEx, CPTAC, HPA databases, and clinical samples. Our findings reveal that NUSAP1 is highly expressed in multiple tumor tissues across most cancer types and is primarily expressed in malignant and immune cells, according to single-cell sequencing data from the TISCH database. Prognostic analysis based on curated survival data from the TCGA database indicates that NUSAP1 expression levels can predict clinical outcomes for 26 cancer types. Furthermore, Gene Set Enrichment Analysis (GSEA) suggests that NUSAP1 promotes cell proliferation, tumor cell invasion, and regulation of anti-tumor response. Analysis of immune score, immune cell infiltration, and anti-cancer immunity cycle using ESTIMATE, TIMER, and TIP databases show that high NUSAP1 levels are associated with low CD4^+^T and NKT cell infiltration but high Th2 and MDSC infiltration, inversely correlated with antigen-presenting molecules and positively correlated with a variety of immune negative regulatory molecules. Notably, patients with melanoma, lung, and kidney cancer with high NUSAP1 expression levels have shorter survival times and lower immunotherapy response rates. Using Cmap analysis, we identify Entinostat and AACOCF3 as potential inhibitors of NUSAP1-mediated pro-oncogenic effects. *In vitro* and *in vivo* experiments further confirm that NUSAP1 knockdown significantly reduces the proliferation ability of A549 and MCF-7 cells. Overall, our study highlights the potential of NUSAP1 expression as a novel biomarker for predicting prognosis and immuno-therapeutic efficacy across different human cancers and suggests its potential for developing novel antitumor drugs or improving immunotherapy.

## Introduction

According to recent data on population-based cancer data reported by the American Cancer Society, cancer is a major public health problem worldwide, with persistently increasing numbers of new cancer cases and cancer deaths each year 1. Compared to conventional and targeted therapy, cancer immunotherapy, based on immune checkpoint blockade, has revolutionized the treatment landscape across multiple tumor types, even as a first-line clinical treatment 2, 3. However, not all tumor patients benefit greatly from or respond to immunotherapy 4. Therefore, it is critical to identify and characterize novel biomarkers for tumor immunotherapy or immunomodulation in order to develop precise immunotherapy strategies and achieve more durable responses.

Nucleolar and spindle-associated protein 1 (NUSAP1), a well-conserved protein in vertebrates, possesses a typical cell cycle-dependent localization and microtubule-binding properties 5. As an important mitotic regulator, NUSAP1 plays an essential role in maintaining the entire process of mitosis, including spindle assembly, chromosome segregation, and cytokinesis 6. Aberrant proliferation with cell cycle dysregulation is a common feature of cancer cells, leading to several studies associating NUSAP1 with various malignant features of human tumors 7-13. NUSAP1 has been found to promote tumorigenesis and the progression of stomach cancer by stabilizing the YAP1 protein 12. High levels of NUSAP1 have been associated with proliferation, invasion, and metastasis in non-small cell lung cancer (NSCLC) 9, pancreatic cancer 8, and breast cancer 11, prostate cancer 13, liver cancer 7. Conversely, low expression of NUSAP1 has been linked to poor prognosis in patients with cervical cancer 10. Moreover, NUSAP1 expression levels seem to be concomitant with the tumor infiltration of various immune cells in breast cancer 14. These findings suggest that NUSAP1 may play different regulatory roles in the progression of human cancers, potentially including the regulation of tumor cell proliferation, migration, and the tumor immune microenvironment. However, there has been a lack of systematically comprehensive pan-cancer studies conducted to explore the predictive value of NUSAP1 for prognosis and immunotherapy response.

In this study, we utilize various public databases and our collected cancer samples to investigate the differential expression levels of NUSAP1 in cancer and normal tissues. Additionally, we characterize the potential function of NUSAP1 and its influence on immune cell infiltration in pan-cancer. Furthermore, we disclose the predictive value of NUSAP1 for tumor prognosis and response to immune checkpoint blockade therapy based on survival data from TCGA and GEO databases. We also conduct a connectivity map analysis to search for candidate compounds that could target the NUSAP1-mediated pro-oncogenic effect. Moreover, we perform *in vitro* cellular experiments to validate the regulatory function of NUSAP1 on cell proliferation using breast cancer and lung adenocarcinoma cell lines. Therefore, our systematical analysis indicates that NUSAP1 could serve a reliable predictive biomarker for prognosis and tumor immunotherapy response. Furthermore, considering that NUSAP1 is a critical microtubule stabilizer 15, 16, the finding from this study may also provide new insights into the development of novel microtubule-based treatments. However, the underlying molecular mechanisms of elevated levels of NUSAP1 in cancer formation would require further basic and clinical research for exploration and understanding.

## Materials and Methods

### mRNA expression analysis of *NUSAP1*

The mRNA expression data of *NUSAP1* in human normal tissues, including bulk and single tissues, was downloaded from the Genotype-Tissue Expression Project (GTEx) database (https://gtexportal.org/home/). To compare the expression levels of *NUSAP1* in normal and 31 pan-cancer tissues of the Cancer Genome Atlas (TCGA) database, the TIMER 2.0 online tool (http://timer.comp-genomics.org/) was utilized. For statistical analysis and graphical presentation of the normalized gene expression data of *NUSAP1* in cancer and normal tissues from TCGA, GTEx, and UCSC Xena databases, the R package UCSCXenaShiny (v1.1.8) was used. As supplementary data, the expression levels of *NUSAP1* in normal and tumor tissues of breast cancer (BRCA), colorectal cancer (Colorectum), liver cancer (LIHC), lung adenocarcinoma (LUAD), prostate cancer (PRAD), and stomach cancer (STAD) were downloaded from the Gene Expression Omnibus (GEO) database and analyzed. The levels of *NUSAP1* transcripts were obtained from the Ensemble website (https://www.ensembl.org/index.html) and the UCSC Xena database, respectively.

### Collation of human samples and immunohistochemical staining

Ten paired normal and tumor tissues were collected from breast and lung adenocarcinoma patients who underwent surgical excision at either Southwest Hospital or Xinqiao Hospital of the Army Medical University, China (Supplementary Table S1). The permission for using human tissue samples in this research has been approved by the Ethics Committees of Southwest Hospital and Xinqiao Hospital, respectively. Tissue samples were processed and analyzed according to the methods described in our previous publications 17, 18, ensuring consistency and comparability experimental procedures. The primary antibody used for detecting NUSAP1 was purchased from Invitrogen (PA5-106697, Invitrogen, CA, USA).

### Protein expression analysis of NUSAP1

The protein levels of NUSPA1 in several cancer types, including breast cancer (BRCA), colon cancer (COAD), glioblastoma (GBM), head and neck squamous cell carcinoma (HNSCC), kidney clear cell carcinoma (KIRC), liver cancer (LIHC), lung adenocarcinoma (LUAD), and ovarian cancer (OV), were analyzed using data from the Clinical Proteomic Tumor Analysis Consortium (CPTAC). For phosphorylation site analysis of NUSAP1 in normal and tumor tissues, the bie tool (http://ualcan.path.uab.edu/index.html) was utilized. Histochemical staining images depicting NUSAP1 in normal and tumor tissues of COAD, GBM, LIHC, OV, pancreatic cancer (PAAD), PRAD, melanoma (SKCM), and STAD were obtained from the Human Protein Atlas database.

### Single-cell expression analysis of *NUSAP1*

The single-cell expression levels of *NUSAP1* in various pan-cancer tissues were analyzed using the Tumor Immune Single-cell Hub (TISCH) database (http://tisch.comp-genomics.org/home/). The expression data of *NUSAP1* mRNA in different cell types of 79 datasets were downloaded and presented graphically using the R package pHeatmap (v1.0.12). In addition, Umap plots showing the expression pattens of *NUSAP1* in different cell types were obtained from the TISCH database.

### Prognostic effect analysis of NUSAP1 in pan-cancer

Expression data of *NUSAP1* and curated survival data of 31 cancer types from the TCGA database were downloaded from UCSC Xena. The data were then matched and analyze. Four types of outcomes, namely overall survival (OS), disease-specific survival (DSS), disease-free interval (DFI), and progression-free interval (DFI) were analyzed to evaluate the prognostic and predictive value of *NUSAP1* levels for each cancer type. Survival analysis was performed using two methods: the Kaplan-Meier method implemented in R packages survival (v3.3-1) and survminer (v0.4.9), and univariate Cox regression analysis using SPSS software (v23.0, IL, USA). The results of analysis were graphically presented using the R package pHeatmap (v1.0.12). For clinical data analysis, expression data of *NUSAP1* and clinical phenotype data were also downloaded from UCSC Xena and matched accordingly. Based on the cutoff value of *NUSAP1* level determined in the OS or DSS analysis, patients in each cancer type were divided into *NUSAP1*-high and -low groups. Related clinical data, including age, sex, T, N, M status, TNM stage, and recurrence status, were compared between these two groups using the chi-square test. Analyzed results were collated and presented graphically using the R package pHeatmap (v1.0.12). To further validate the prognostic value of *NUSAP1*, outcomes analysis of GEO datasets was performed. Kaplan-Meier plots were generated using the KM plot online tool 19 to analyze OS, post-progression survival (PPS), recurrence-free survival (RFS), progression-free survival (PFS), first progression (FP), DSS, and distant metastases-free survival (DMFS) of patients with BRCA, LIHC, LUAD, OV, and STAD.

### Gene Set Enrichment Analysis (GSEA)

The TCGA patients in each cancer type were divided into high and low groups based on the expression level of* NUSAP1*, and the differentially expressed genes (DEG) between these two groups were used for GSEA. The cancer-related hallmark gene set file (h.all.v7.5.1.symbols.gmt) was downloaded from the Molecular Signatures Database (https://www.gsea-msigdb.org/gsea/msigdb). Normalized Enrichment Score (NES) and False Discovery Rate (FDR) were calculated using the DEGs utilizing the hallmark gene set, utilizing the R package GSEA (v1.38.2). The analyzed results were summarized and graphically presented in the bubble plot using the R package ggplot2 (v3.3.6).

### Immune score and immune cell infiltration analysis

Immune score data for each patient in 18 cancer types were downloaded from the ESTIMATE database (https://bioinformatics.mdanderson.org/estimate/). The immune score between *NUSAP1*-high and -low patients (based on cutoff value of the OS or DSS analysis) was compared and graphically presented using the R package ggplot2 (v3.3.6). To analyze the influence of *NUSAP1* expression and mutant status on immune cell infiltration in 40 cancer types, the TIMER 2.0 database was utilized. The correlation between *NUSAP1* expression and infiltration degree of 15 immune cell types, including CD4^+^T, CD8^+^T, B, follicular helper T cell (TFH), dendritic cell (DC), macrophage, regulatory T cell (Treg), natural killer (NK), NKT, γδT, myeloid-derived suppressor cell (MDSC), monocyte (Mono), MAST, neutrophils (Neut), and eosinophils (Eosi), two stromal cells (carcinoma-associated fibroblast (CAF) and endothelial cell), and three progenitor types was explored using spearman correlation analysis.

### Anti-cancer immune response analysis

The impact of *NUSAP1* expression level on the status of anti-cancer immunity was analyzed in 22 cancer types using the Tracking Tumor Immunophenotype (TIP) database (http://biocc.hrbmu.edu.cn/TIP) 19. Immune activity scores between *NUSAP1*-high and -low samples were compared using either Student's t test or Mann-Whitney U test. The analyzed results for the 22 cancer types were summarized and graphically presented using the R package pHeatmap (v1.0.12) and ggplot2 (v3.3.6).

### Predictive analysis of immunotherapy response

The expression correlation between *NUSAP1* and 75 immune-related regulators in 31 cancer types was analyzed using cor.test function in RStudio (v1.3.1093) with TCGA expression data. The results were summarized and presented visually using the R package pHeatmap (v1.0.12). Moreover, the correlation between *NUSAP1* expression and tumor mutation burden (TMB) or microsatellite instability (MSI) was analyzed using UCSCXenaTools (v1.4.7) online tool (https://shiny.hiplot.com.cn/ucsc-xena-shiny) 20. *NUSAP1* expression and related pathological data from anti-PD-1/PDL-1-treated LUAD patients, anti-PD-L1-treated kidney cancer patients, and anti-PD-1-treated melanoma patients were obtained from the GSE135222, IMvigor210, and GSE91061 datasets, respectively. Survival analysis was performed using the survival (v3.3-1) and survminer (v0.4.9) packages in R.

### Connectivity Map (Cmap) analysis

Differentially expressed genes between *NUSAP1*-high and -low samples in each cancer type were obtained from the previous GSVA analysis in this study. The 500 most upregulated or downregulated genes were collected and utilized as the *NUSAP1*-related signature. The CMAP_gene_signatures. RData file, which contains 1288 compounds-related signatures, was downloaded from the database website (https://www.pmgenomics.ca/bhklab/sites/default/files/downloads), and used for calculating the matching score. The analysis process was followed the methodology outlined in previous publications 21, 22. The results for 31 cancer types were summarized and presented graphically using the package pHeatmap (v1.0.12) in R.

### Construction of cell lines stably expressing *NUSAP1 shRNA*

HEK293, MCF-7, and A549 cell lines were purchased from the American Type Culture Collection (ATCC). *NuSAP1* shRNA was obtained from clone ID: TRCN0000136422. The *NUSAP1* targeting sequence was 5'-CCTCAGGTAACAGAGATTC-3' and the control shRNA sequence was 5'- GCAGTTATCTGGAAGATCAGG-3'. The *NUSAP1* and control shRNA sequence were subcloned into the pLV-H1-EF1α-puro vector, respectively. To package virus, HEK293 cells were transfected with the pLV-H1-EF1α-puro-NUSAP1/control shRNA plasmid, along with Gag-Pol, Rev, and VSV-G plasmids, in a ratio of 1:0.45:0.18:0.27. The transfection was carried out for 72 hours. Subsequently, MCF-7 and A549 cells were infected with NUSAP1/control shRNA viruses for 48h in the presence of 8 μg/mL of polybrene (Sigma, MO, USA). Stable A549 and MCF-7 cells were generated under the selection of 2 μg/mL puromycin (Sigma). The efficiency of interference was assessed by western blotting with 1:1000 dilution of rabbit anti-NUSAP1 (#12024-1-AP, Proteintech, IL, USA) and mouse anti-GAPDH antibodies (#AC002, ABclonal, MA, USA).

#### MTS assay and cell counting

MTS assay and cell counting were performed to assess the impact of NUSAP1 on the cell viability and proliferation ability of A549 and MCF-7 cells. For MTS assays, the CellTiter 96 AQueous One Solution Cell Proliferation Assay system (Promega) was used according to the manufacturer's instructions. A total of 1 × 10^4^ cells were plated in each well of a 96-well plate and 20 μL of CellTiter 96 AQueous One Solution reagent was added to each well containing 100 μL of medium. After 1 hour incubation in humidified 5% CO_2_ incubator, the absorbance at 490 nm was measured using an Hidex Sense microplate reader. For the proliferation experiment, 5 x 10^4^ cells were seeded in 24-well plates, and the cell count was determined at 24h, 48h, 72h, respectively.

#### Colony formation

To evaluate the impact of NUSAP1 on long-term proliferation ability, 1 × 10^3^ control or *NUSAP1-*knockdown A549 and MCF-7 cells were seeded in six-well plates. The medium was replaced every three days to maintain the cell's normal growth state. The cells were then cultured for 14 days, and then the colonies were washed with PBS, fixed in 4% paraformaldehyde for 15 mins, and stained with crystal violet (0.1%) for 30-40 min. Finally, colonies were photographed and counted.

#### Subcutaneous xenograft model

All mice experiments were conducted in accordance with the protocols approved by the Ethical Committee for Animal Experimentation of the Army Medical University. SPF grade female nude mice (5 weeks old with a mean body weight of 20 g) were purchased from Vital River Laboratory Animal Technology (Beijing, China). To evaluate the effect of NUSAP1 on the cell proliferation ability *in vivo*, 1 × 10^7^ control or *NUSAP1-*knockdown A549 and MCF-7 cells were subcutaneously inoculated in the underarm region of the right foreleg of each mouse (5 mice for per group). The tumor diameter and weight of tumor-bearing mice were measured at 3, 6, 9, 12, 16, 20, and 22 days respectively after inoculated. Tumor volume was calculated using the formula: volume = (long diameter) × (short diameter)^2^/2. After 20 days, the mice were euthanized, and the tumors were isolated, weighted, photographed. The tumors were then lysed and total protein was extracted for western blotting analysis to assess the levels of NUSAP1 and KI67 proteins in each tumor. Antibodies used for western blotting was rabbit anti-NUSAP1 (PA5-106697, Invitrogen) at a 1:1000 dilution, rabbit anti-KI67 (MA5-14520, Invitrogen) at a 1:1000 dilution, and mouse anti-β-Actin (3700S, CST, MA, USA) at a 1:20000 dilution.

### Statistical Analysis

Statistical analysis in this study was performed using SPSS (v23.0, NY, USA) and Graphpad Prism (v8.0.1, CA, USA) software. For comparisons of continuous variables, Student's t-test for two groups was used if the data were normally distributed, and the Mann-Whitney U test was used when the data were not normally distributed. Chi-square and Fisher's exact test were used for comparisons of categorical data. Univariate Cox regression analysis and Kaplan-Meier method were employed to assess the prognostic value of *NUSAP1* level. Spearman correlation analysis was employed to analyze the expression correlation between *NUSAP1* and immune-related regulators. Statistical significance was set at P < 0.05.

## Results

### Expression pattens of *NUSAP1* in normal and cancer tissues

**Figure 1:**
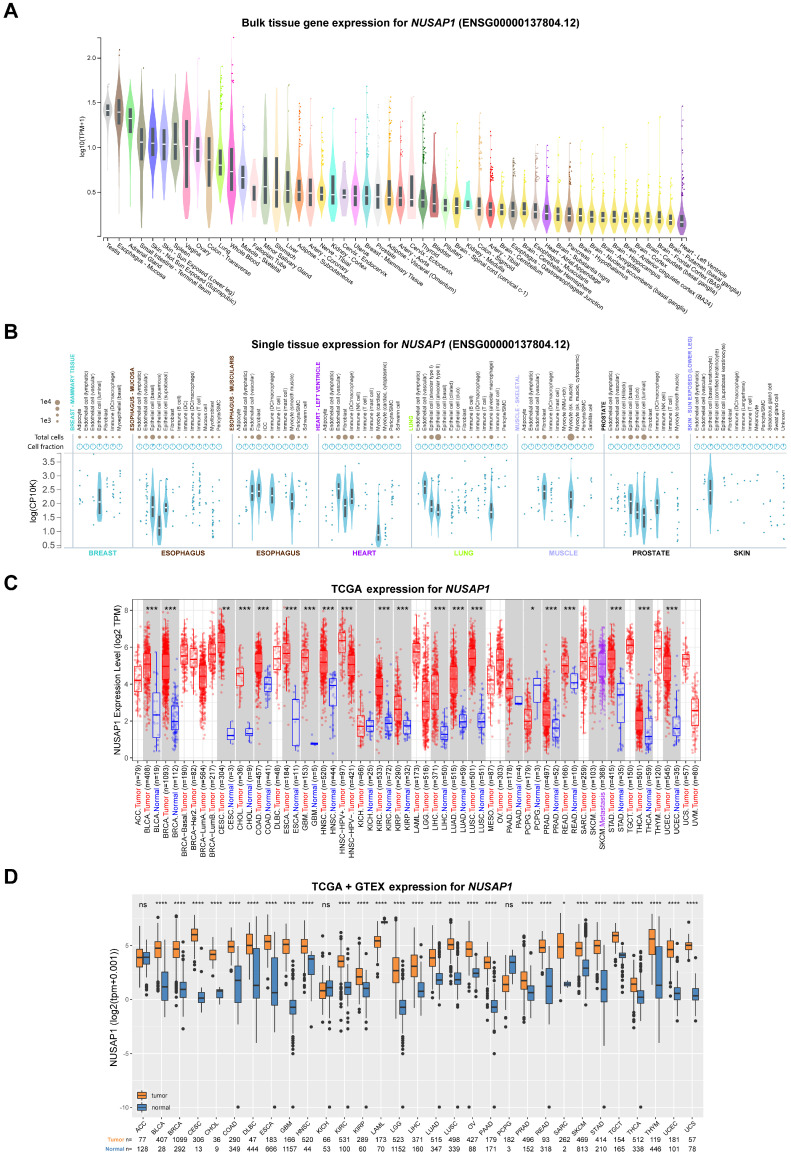
** mRNA expression levels of *NUSAP1* in human normal and tumor tissues. (A)** Violin plots showing *NUSAP1* expression levels in various human normal tissues.** (B)** Violin plots displaying the single-cell expression data of *NUSAP1* in human normal breast, esophagus, heart, lung, muscle, prostate and skin tissues. **(C)** Boxplots illustrating the mRNA expression levels of *NUSAP1* in normal and cancer tissues using data from the TCGA database. Tumor tissues are represented by red dots and boxes, while normal tissues are represented by blue dots and boxes. **(D)** Boxplots showing the mRNA expression levels of *NUSAP1* in normal and cancer tissues using data from the TCGA database. Tumor tissues are represented by orange boxes, and normal tissues are represented by blue boxes. The symbols ns, *, **, and *** indicate not significant, P < 0.05, P < 0.01, P < 0.001, respectively.

To investigate the basic expression levels of *NUSAP1* in human normal tissues, we employed the GTEx database. As shown in Figure 1A, relatedly higher mRNA levels of *NUSAP1* were found in testis and numerous organs, including esophagus, adrenal gland, skin, ovary, colon, lung, stomach, liver, kidney, cervix, breast, and prostate, which are known to be susceptible to cancer development. In contrast, *NUSAP1* was expressed at low levels in non-proliferative tissues such as heart and brain (Figure 1A). Furthermore, the results from single-cell RNA sequencing showed that high expression of *NUSAP1* in various epithelial cells in normal breast, esophagus, lung, prostate, and skin tissues (Figure 1B). To compare the mRNA levels of *NUSAP1* between cancer and normal tissues, we downloaded expression data from the TCGA and GTEx databases. The analyzed results uncovered significantly higher *NUSAP1* expression in almost all cancer tissues compared to normal tissues, except for ACC, KICH, and PCPG (Figure 1C and 1D). Consistently, the elevated expressions of *NUSAP1* in most cancers were also validated in the GEO datasets (Supplementary Figure 1).

**Figure 2:**
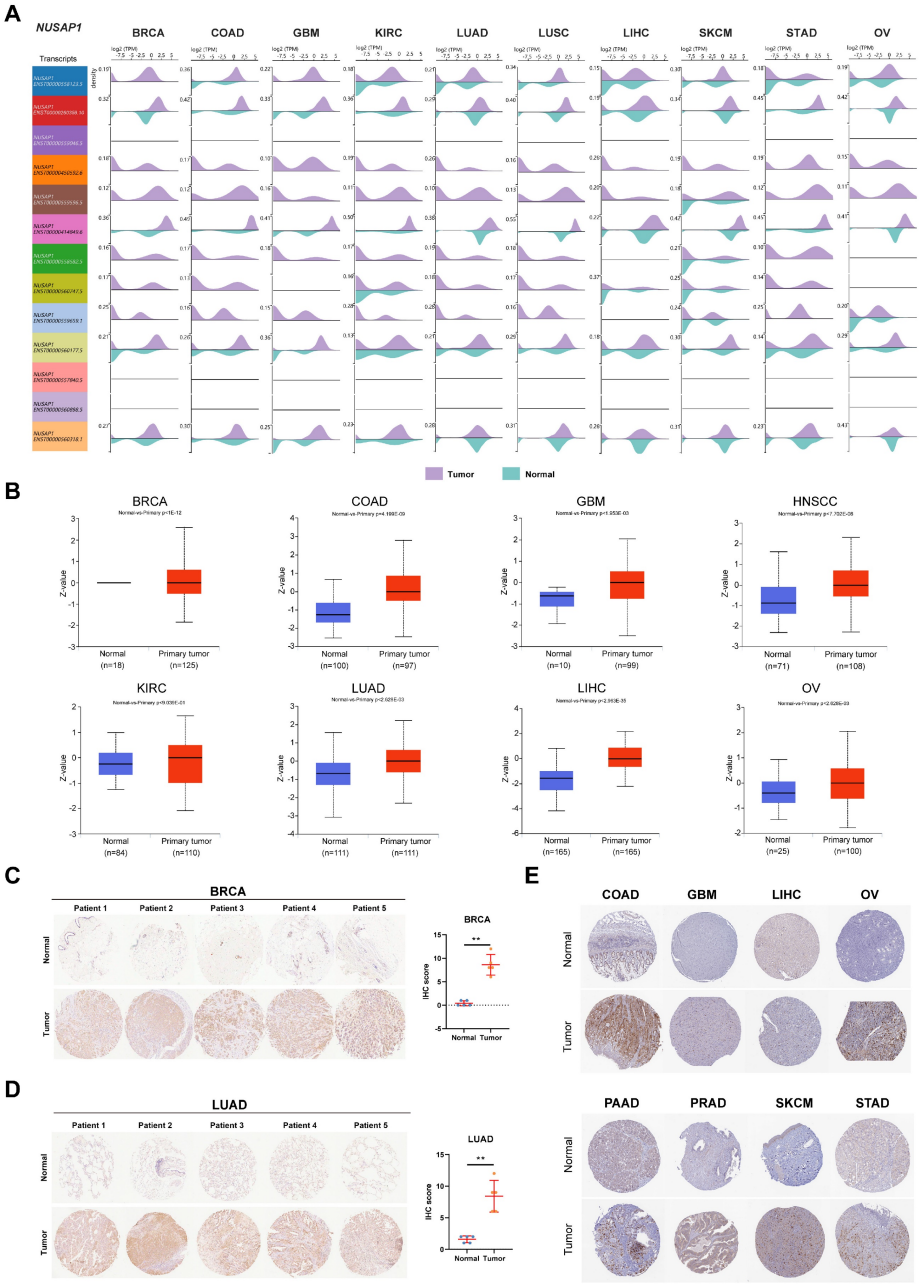
** Differential expression of NUSAP1 transcripts and protein levels in normal and tumor tissues. (A)** Bean plots showing the expression levels of 13 transcripts of *NUSAP1* in normal and cancer tissues. Purple color represents tumor tissues, while lake green boxes represent normal tissues. **(B)** Boxplots indicating the protein levels of NUSAP1 in normal and cancer tissues. Blue boxes represent normal tissues, and red boxes represent tumor tissues. **(C-D)** Histochemical staining results of NUSAP1 protein in 5 paired normal and tumor tissues from patients with BRCA **(C)** and LUAD **(D)** (left). Scatter plots (right) showing the IHC score for each sample. Blue dots represent normal tissues, and yellow dots represent tumor tissues. **(E)** Representative images of immunohistochemical staining of NUSAP1 in 8 types of normal and tumor tissues. The symbol ** indicates statistical significance at P < 0.01.

To investigate the change in mRNA levels, we examined the differential expression of *NUSAP1* transcripts in normal and cancer tissues. The *NUSAP1* gene, located at chromosome 15, generates 13 distinct transcripts (Supplementary Figure 2A). Among these two transcripts, two protein-coding transcripts, ENST00000450592.6 (colored in orange; row #4) and ENST00000559596.5 (colored in brown; row #5), were obviously elevated in tumor tissues, compared to other transcripts (Figure 2A). Moreover, utilizing the UALCAN database, we further conducted an analysis of NUSAP1 protein levels between normal and primary tumor tissues. As shown in Figure 2B, NUSAP1 protein was significantly upregulated in LUAD, BRCA, LICH, GBM, OV, KIRC, COAD, and HNSC tissues. Consistently, through histochemical staining, we confirmed the elevated protein levels of NUSAP1 in BRCA and LUAD cancer, but not normal, using our collected clinical individual patient samples (Figure 2C and 2D). The HPA database also confirmed similar expression patterns of NUSAP1 protein in other cancer types (Figure 2E). To predict the potential signaling regulation in cancers, we examined the phosphorylation sites of NUSAP1 using the CPTAC database. Interestingly, these results suggested that phosphorylation of NUSAP1 at Ser309 and Thr312, located in the microtubule-associated domain, was significantly elevated in HNSC tumor tissue compared with normal tissue (Supplementary Figure 2B and 2C). This effect leads to the inhibition of NUSAP1 binding to microbundle during early mitosis, resulting in slower cell proliferation 23. Taken together, these findings suggest that NUSAP1 may play an important role in the process of tumorigenesis and tumor development.

### Single-cell expression levels of *NUSAP1* in multiple cancer tissues

**Figure 3:**
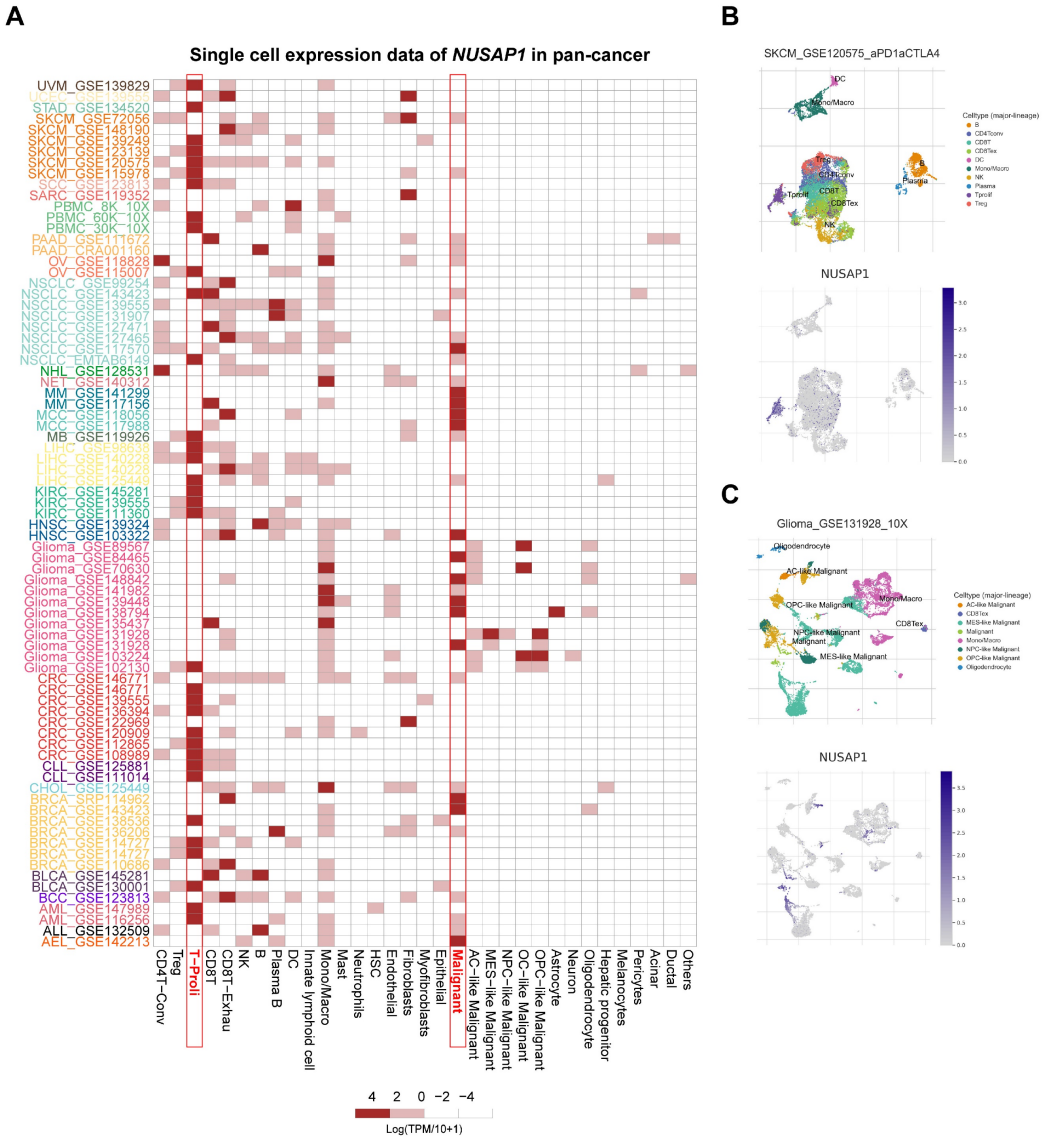
** Single-cell expression analysis of *NUSAP1* in tumor tissues. (A)** Cluster heatmaps showing the mRNA levels of *NUSAP1* in 33 cell types of tumor tissues. **(B-C)** Umap plots displaying the clustering of different cell types (*upper panel*) and *NUSAP1* expression level (*lower panel*) in SKCM **(B)** and Glioma **(C)** tissues.

To further investigate the cell types express *NUSAP1* in tumor tissues, we analyzed the single-cell expression of *NUSAP1* using 79 datasets from the TISCH database. As shown in Figure 3A, the heatmap, illustrating the relative expression levels of *NUSAP1* across 33 cell types, indicated widespread expression of *NUSAP1* in various immune and malignant cells. For instance, UMAP plots revealed the expression of *NUSAP1* in CD4^+^T, CD8^+^T, NK, B, DC, monocyte, and macrophage in melanoma (SKCM_GSE120575), with particularly high expression observed in proliferating T cell (T-proli) (Figure 3B, the lower plot). It is worth pointing out that in glioma (Glioma_GSE131928), *NUSAP1* was predominantly expressed in malignant cells, monocyte, and macrophage, but not in T-proli (Figure 3C). Furthermore, *NUSAP1* exhibited an inverse expression pattern between T-proli and exhausted CD8^+^T cells in multiple cancer tissues, suggesting its potential regulatory role in T cell function.

### Prognostic role of NUSAP1 in human cancers

**Figure 4:**
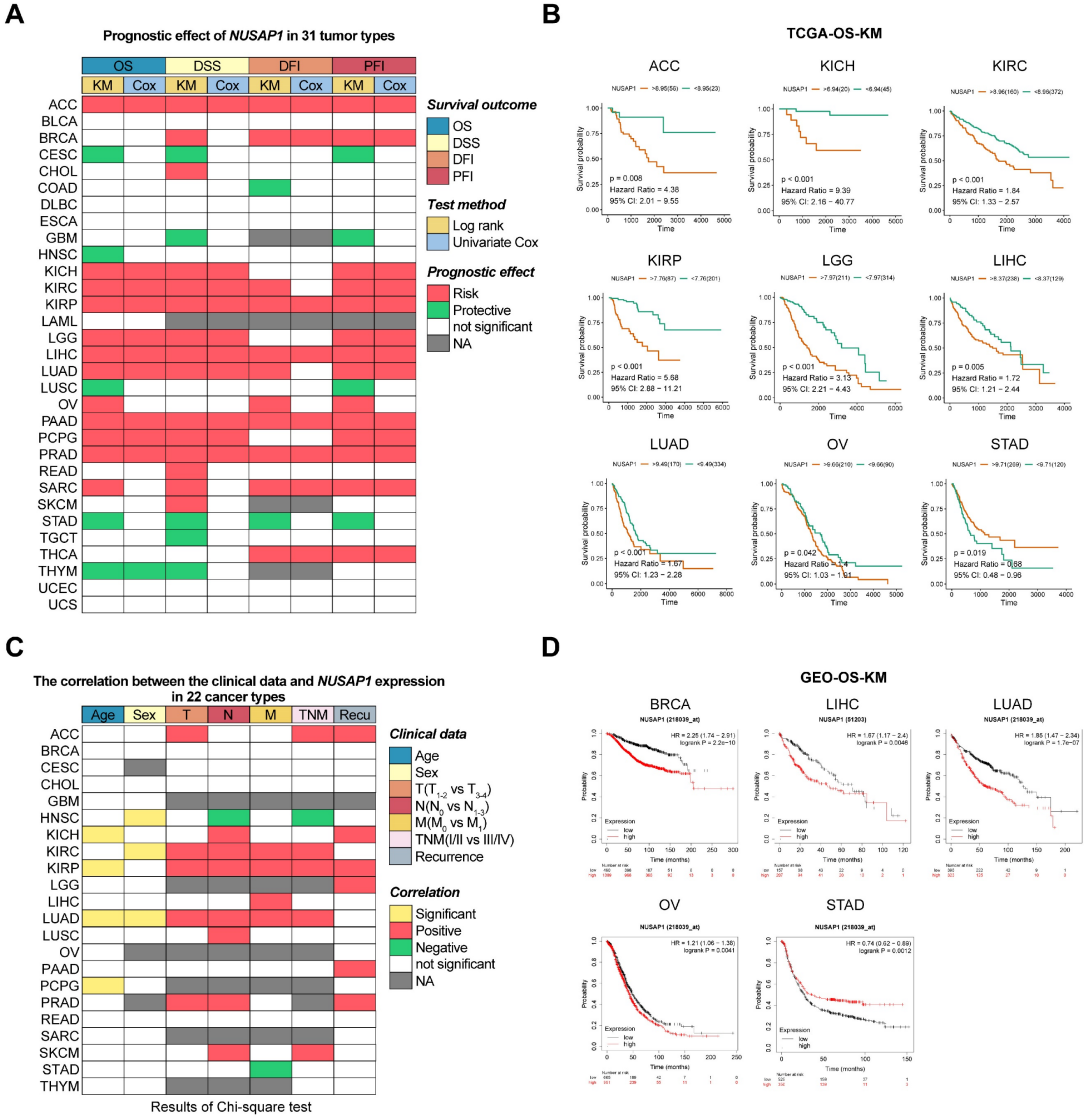
** Predictive effects of *NUSAP1* expression levels on prognosis in human cancers. (A)** Heatmap showing the correlation between *NUSAP1* expression levels and four curated survival outcomes, including overall survival (OS), disease-specific survival (DSS), disease-free interval (DFI) and progression-free interval (PFI). Survival analysis was performed using a log rank (KM) test and univariate Cox regression, based on curated survival data from the TCGA database. Red boxes represent a risk factor, green boxes represent a protective factor, white boxes represent the analyses are not significant, and gray boxes represent the data are not available. **(B)** Representative survival curves of prognostic analysis comparing *NUSAP1*-high and *NUSAP1*-low patients in ACC, KICH, KIRC, KIRP, LGG, LIHC, LUAD, OV, and STAD. **(C)** Heatmap clusters showing the correlation between *NUSAP1* expression levels and seven clinical characteristics in 22 cancer types. Data were downloaded from the TCGA database and analyzed using the Chi-square test. A cutoff value in OS or DSS analysis was used to differentiate between high and low *NUSAP1* expressions. Yellow boxes represent significant differences in correlation with age or sex, red and green boxes represent more advanced and low-grade clinical characteristics in high *NUSAP1* patients, white boxes indicate non-significant results and gray boxes represent unavailable data. **(D)** Representative survival curves of prognostic analysis comparing *NUSAP1*-high and -low patients in BRCA, LIHC, LUAD, OV, and STAD. Data were analyzed using the KM plot online tool with GEO datasets.

To investigate the prognostic impact of *NUSAP1* mRNA levels in human cancers, we downloaded and analyzed curated survival data from the UCSC database, including OS, DSS, DFI, and PFI for 31 different cancer types. Heatmap clustering results showed that the expression level of *NUSAP1* was a predictive factor for prognosis in patients in the 25 tumor types, excluding BLCA, DLBC, ESCA, LAML, UCEC, and UCS (Figure 4A and Supplementary Table S2). Further analysis showed that relatively higher expression levels of *NUSAP1* were significantly associated with both poor OS and DSS in patients with ACC, KICH, KIRC, KIRP, LGG, LIHC, LUAD, PAAD, PCPG, PRAD, and SARC. Conversely, high levels of *NUSAP1* were correlated with good prognosis in patients with CESC, GBM, HNSC, LUSC, STAD, TGCT, THYM (Figure 4B and Supplementary Figure S3). These findings were further supported by analysis of DFI and PFI (Figure 4A; last 4 columns). Interestingly, the analysis of these prognostic outcomes indicated that NUSAP1 could be a protective factor for gastric cancer.

To gain a better understanding of the association between *NUSAP1* expression and clinical features in these cancers, we downloaded and analyzed clinical phenotype data of TCGA. Significant differences in *NUSAP1* expression levels were observed age groups, under 45 and over 45, in KICH, KIRP, LUAD, and PCPG patients, as well as between males and females in HNSC, KIRC, and LUAD patients. High *NUSAP1* expression was associated with increased proliferation and invasion (T status) in ACC, KIRC, KIRP, LUAD, and PARD. Additionally, patients with high *NUSAP1* expression in KICH, KIRC, KIRP, LUAD, LUSC, PARD and SKCM were more prone to lymph node metastasis (N status), and *NUSAP1* levels positively correlated with distant metastases (M status) in patients with KIRC, KIRP, LIHC, and LUAD. *NUSAP1*-high patients with ACC, KIRC, KIRP, LUAD, and SKCM also showed a significant trend towards higher TNM stages. Furthermore, patients with high *NUSAP1* expression in ACC, KICH, KIRP, LGG, PAAD, and PARD had a higher risk of recurrence compared to those with low* NUSAP1* levels (Figure 4C and Supplementary Table S3).

Subsequently, to further validate the impact of *NUSAP1* expression on tumor prognosis, we utilized multiple GEO datasets. KM survival analysis consistently demonstrated that patients with high levels of *NUSAP1* in BRCA, LIHC, LUAD, and OV had poor overall OS, PPS, RFS, PFS, DSS, and FP. Similarly, high *NUSAP1* levels were consistently a protective factor for OS and PPS in STAD patients (Figure 4D and Supplementary Figure S4). Taken together, these results demonstrate that the expression level of *NUSAP1* is an effective prognostic factor for multiple cancers.

### GSEA analysis of potential functions of NUSAP1 in human cancers

**Figure 5:**
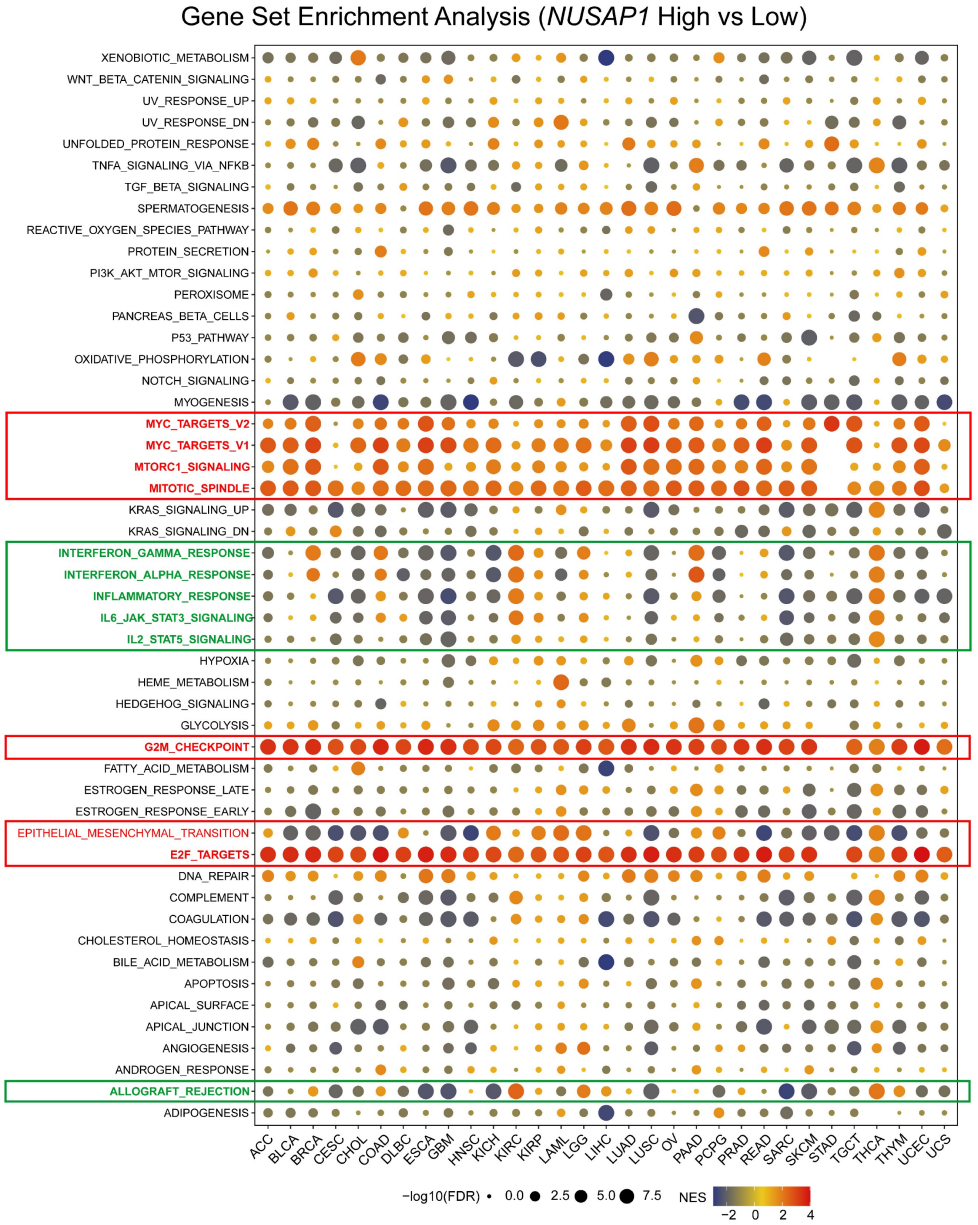
** Potential function analysis of NUSAP1 in human cancers using GSEA.** A bubble plot shows the results of GSEA between *NUSAP1*-high and -low tumor patients using hallmark gene signatures. The size of each circle represents the magnitude of the P value, while the color codes from red to yellow to blue represents the magnitude of the normalized enrichment scores (NES).

To gain further insight into the impact of NUSAP1 on tumor patient prognosis, a pan-cancer Gene Set Enrichment Analysis (GSEA) was conducted. This analysis examined differentially expressed genes between *NUSAP1*-high and -low patients in each cancer type. The resulting heatmap revealed significant enrichment of cell proliferation-related signaling pathways, including MYC, mTORC1, Mitotic spindle, G2M, and E2F pathways, in the *NUSAP1*-high patients across almost all cancer types (Figure 5). This finding aligns with a previous report suggesting that NUSAP1 promotes the organization of mitotic spindle microtubules around chromosomes, indicating its crucial role in regulating cell proliferation (5).

In addition, high expression of *NUSAP1* exhibited a positive correlation with epithelial-mesenchymal transition (EMT) in ACC, KICH, KIRC, KIRP, LGG, and PAAD patients. This correlation may explain why patients with high *NUSAP1* levels in these cancer types are prone to lymph node and distant metastasis, as well as recurrence. Interestingly, the enrichment of the EMT pathway was not significant in HNSC and STAD patients with high *NUSAP1* levels, consistent with the analysis of N and M status in these two cancers (Figure 4C and 5).

Moreover, the heatmap clusters also highlighted the differential enrichment of immune-related pathways, such as interferon (IFN)-gamma, IFN-α, inflammatory, IL-6, IL-12, complement, and allograft rejection pathways (Figure 5). These pathways were negatively enriched in most cancer patients with a high expression level of *NUSAP1*, including CESC, CHOL, GBM, KICH, LUSC, PCPG, SARC, SKCM, and THYM. These findings suggest that NUSAP1 may be involved in suppressing the antitumor immune response in these cancers. Notably, for LUAD patients, high-levels of *NUSAP1* were also inversely enriched with immune response, IL-6, IL-12, and complement pathway, albeit to a moderate extent (Figure 5). In conclusion, these results strongly suggest that elevated levels of *NUSAP1* are tightly associated with increased proliferation, EMT, and immunosuppression in human cancers.

### Effect of *NUSAP1* expression and mutations on immune cell infiltration in human cancers

**Figure 6:**
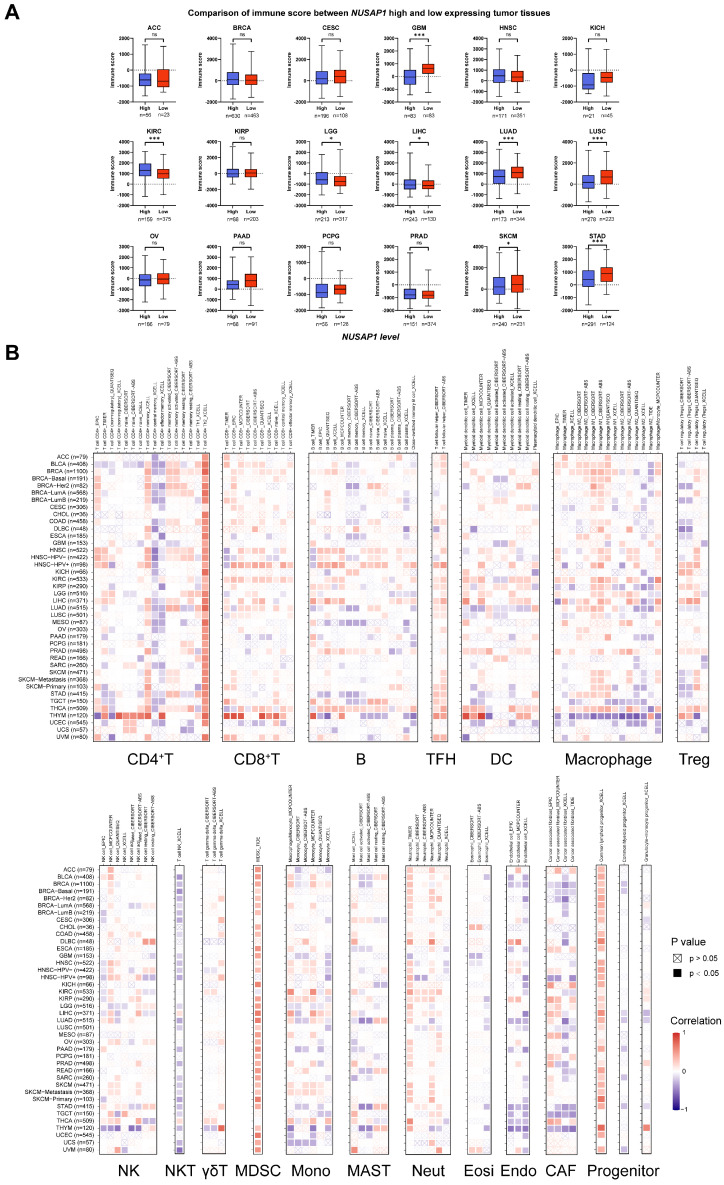
** Correlation analysis between *NUSAP1* expression and immune cell infiltration. (A)** Boxplots show the comparison of immune scores between *NUSAP1*-high and -low patients, distinguished by a cutoff value in OS or DSS analysis. The red dots and boxes represent the patients with high levels of *NUSAP1*, while the lake-blue dots and boxes represent the patients with low levels of *NUSAP1*. **(B)** Cluster heatmaps display the correlation between *NUSAP1* expressions and the degree of infiltration by CD4^+^T, CD8^+^T, B, TFH, DC, macrophage, Treg, NK, NKT, γδT, MDSC, monocyte (Mono), MAST, neutrophil (Neut), eosinophil (Eosi), endothelial (Endo), cancer-associated fibroblast (CAF), and progenitors infiltration. Data were analyzed using TIMER online tools. The red squares represent a positive correlation, while blue squares represent a negative correlation. Empty boxes with a cross indicate a nonsignificance correlation (P > 0.05) and black boxes indicate a significance correlation (P < 0.05).

To investigate whether *NUSAP1* expression can influence the immune cell infiltration in human cancers, we first analyzed the ESTIMATE database to compare immune scores between *NUSAP1*-high and *NUSAP1*-low expressing patients in 18 cancer types. The immune scores of the high *NUSAP1* expression in GBM, LUAD, LUSC, SKCM, and STAD were significantly lower than that of patients with low *NUSAP1* expression. However, the immune scores appeared to be positively correlated with *NUSAP1* levels in KIRC and LGG patients (Figure 6A). These findings were consistent with that the results from GSEA (Figure 5), indicating that NUSAP1 may inhibit immune cell infiltration in GBM, LUAD, LUSC, SKCM, and STAD, while enhancing cell infiltration process in KIRC and LGG.

To explore which immune cell types could be influenced by *NUSAP1* expression in pan-cancer, Spearman correlation analyses were performed utilizing data from the TIMER 2.0 database. As shown in Figure 6B, *NUSAP1* expression was positively associated with the infiltration of Th2, TFH, MDSC, neutrophil, and lymphoid progenitor cells, but negatively associated with central and effector memory CD4^+^T, NKT, and endothelial cells. The increase in Th2 and MDSC cells and the decrease of memory CD4^+^T and NKT in the tumor may indicate the suppression of the immune microenvironment (TME) 24.

In addition to *NUSAP1* expression levels, we also observed the effect of *NUSAP1* mutations on the infiltration of six immune cells (B, CD8^+^T, CD4^+^T, macrophages, neutrophils, and dendritic cells) into the TME of 31 cancer types. The results revealed that both four different types of* NUSAP1* gene mutations, including deep deletion, arm-level deletion, arm-level gain, and high amplification, could decrease the infiltration of six immune cells in most cancer types (Supplementary Figure S5). These findings indicate that the expression of *NUSAP1* in tumor cells could be involved in regulating the migration and infiltration of immune cells, thereby impacting the prognosis and immunotherapy of human cancers.

### Predictive potential of NUSAP1 in cancer immunotherapy response

**Figure 7:**
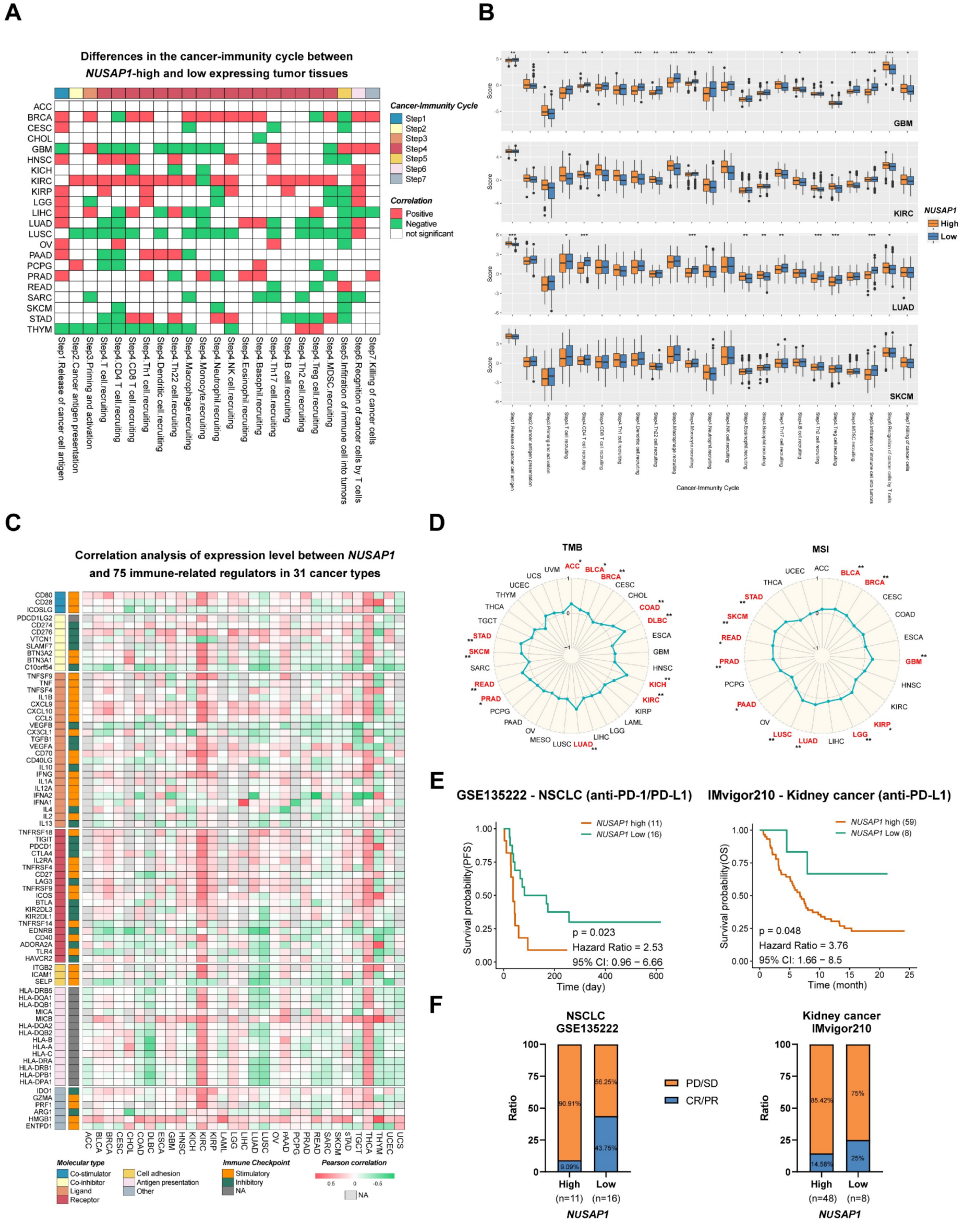
** Influence of *NUSAP1* expression on anti-tumor immunity and immunotherapy response. (A)** Heatmap clusters show the differences in the cancer-immunity cycle between *NUSAP1*-high and -low expressing tumor tissues, distinguished by a value in OS or DSS analysis. The red boxes represent a positive correlation, the green boxes represent a negative correlation, and the white boxes represent nonsignificant correlations. **(B)** Representative boxplots display the correlation analysis of the cancer-immunity cycle in GBM, KIRC, LUAD, and SKCM. The orange boxes represent the patients, with high levels of *NUSAP1*, while blue boxes represent the patients with low levels of *NUSAP1*. **(C)** Cluster heatmaps show the correlation analysis of the expression levels between *NUSAP1* and 75 immune genes in 31 cancer types. The red boxes represent a positive correlation, the green boxes represent a negative correlation, and the light gray boxes represent the data were not available. **(D)** Radar plots demonstrate the correlation between *NUSAP1* expression and tumor mutation burden (TMB, *left*) and microsatellite instability (MSI, *right*) of multiple tumor types. **(E)** Predictive values of *NUSAP1* expression on PFS and OS of NSCLC (*left*) and kidney cancer (*right*) patients in anti-PD-1/PD-L1 immunotherapy.** (F)** Response rate of immunotherapy in NLSCL (*left*) and kidney (*right*) cancer patients. PD, progressive disease; SD, stable disease; CR, complete response, and PR refers to partial response.

Given the results of our previous analysis, which demonstrated the regulatory role of *NUSAP1* expression levels in immune responses and immune cell infiltration in various human cancers, we proceeded to examine the predictive effect of NUSAP1 on cancer immunotherapy. To assess this, activity scores of the cancer-immunity cycles from the TIP database were downloaded and evaluated. As shown in Figure 7A and Supplementary Table S4, high *NUSAP1* expression was positively correlated with the release of cancer cell antigen (Step 1), recruitment of Th1 cell (Step 4) and recognition of cancer cell by T cells (Step 6). However, a negative correlation was observed between high *NUSAP1* levels and immune cell infiltration into tumors (Step 5) in most cancer types, further indicating the immunosuppressive effect of NUSAP1 in TME.

To delve deeper into the analysis, GBM, LUAD, LUSC, SARC, and SKCM patients with high levels of *NUSAP1* expression exhibited lower immune activity scores in most steps compared to those with low *NUSAP1* expression, including T and CD4^+^T cell recruiting (Step 4). Additionally, a positive association was observed between activity scores of Th2, Treg, MDSC recruitment (Step 4) and *NUSAP1* levels in KIRC (Figure 7B).

Immune-related regulators plays a crucial role in modulating the tumor microenvironment and influencing the efficiency of cancer immunotherapy 25. To investigate the association of *NUSAP1* with 75 immune-related molecules across 31 cancer types, a heatmap analysis was conducted. The results revealed a negative correlation between *NUSAP1* and antigen presentation-related molecules (except MICB) in most cancer types, However, in KIRC and THCA, *NUSAP1* exhibited a positive correlation with the majority of immune-related molecules. Notably, high expression levels of *NUSAP1* were positively correlated with various immune checkpoint molecules, including CD274 (PD-L1), CD276, TIGHT, PDCD1 (PD-1), CTLA4, LAG3, IDO1 in BRCA, GBM, KICH, KIRC, KIRP, LGG, PAAD, LUAD, STAD, and THCA. Furthermore, VEGFA 26, known to hinder tumor immunotherapy, demonstrated a positive correlation with the expression levels of *NUSAP1* in GBM, KICH, KIRC, KIRP, LUAD, PAAD, SARC, and SKCM. Similarly, evaluated levels of TGF-β1 was also observed in *NUSAP1* highly expressing patients with GBM, KICH, KIRC, KIRP, LGG, and THCA (Figure 7C and Supplementary Table S5) 27.

Considering the significance of PD-L1, TMB and MSI as important biomarkers for immunotherapy, the correlation between TMB/MSI and *NUSAP1* expression was also assessed across multiple cancer types. The results demonstrated a positive correlated between *NUSAP1* expression and high TMB scores in ACC, BLCA, BRCA, COAD, KICH, KIRC, LUAD, PRAD, READ, SKCM, and STAD. Similarly, high MSI scores were positively associated between *NUSAP1* expression in BLCA, BRCA, GBM, KIRP, LGG, LUAD, LUSC, PRAD, READ, SKCM, and STAD (Figure 7D). These results suggest that NUSAP1 has the potential to serve as a predictive marker for the efficiency of cancer immunotherapy in the corresponding cancers.

Subsequently, we proceeded to analyze the prognostic value of *NUSAP1* expression as a predictor for immune checkpoint blockade therapy. Survival analysis showed that patients with low *NUSAP1* levels exhibited improved PFS in NSCLC) and OS in kidney cancer and SKCM when treated with anti-PD-1/PD-L1 therapy, compared to those with high *NUSAP1* levels (Figure 7E and Supplementary Figure S6). Furthermore, NSCLC and kidney cancer patients with high *NUSAP1* expression had lower response rates to immunotherapy (Figure 7F). These findings solidify the predictive potential of NUSAP1 in immunotherapy response and suggest its promising utility as a biomarker for cancer immunotherapy.

### CMap analysis identifies potential compounds targeting NUSAP1 in pan-cancer

**Figure 8:**
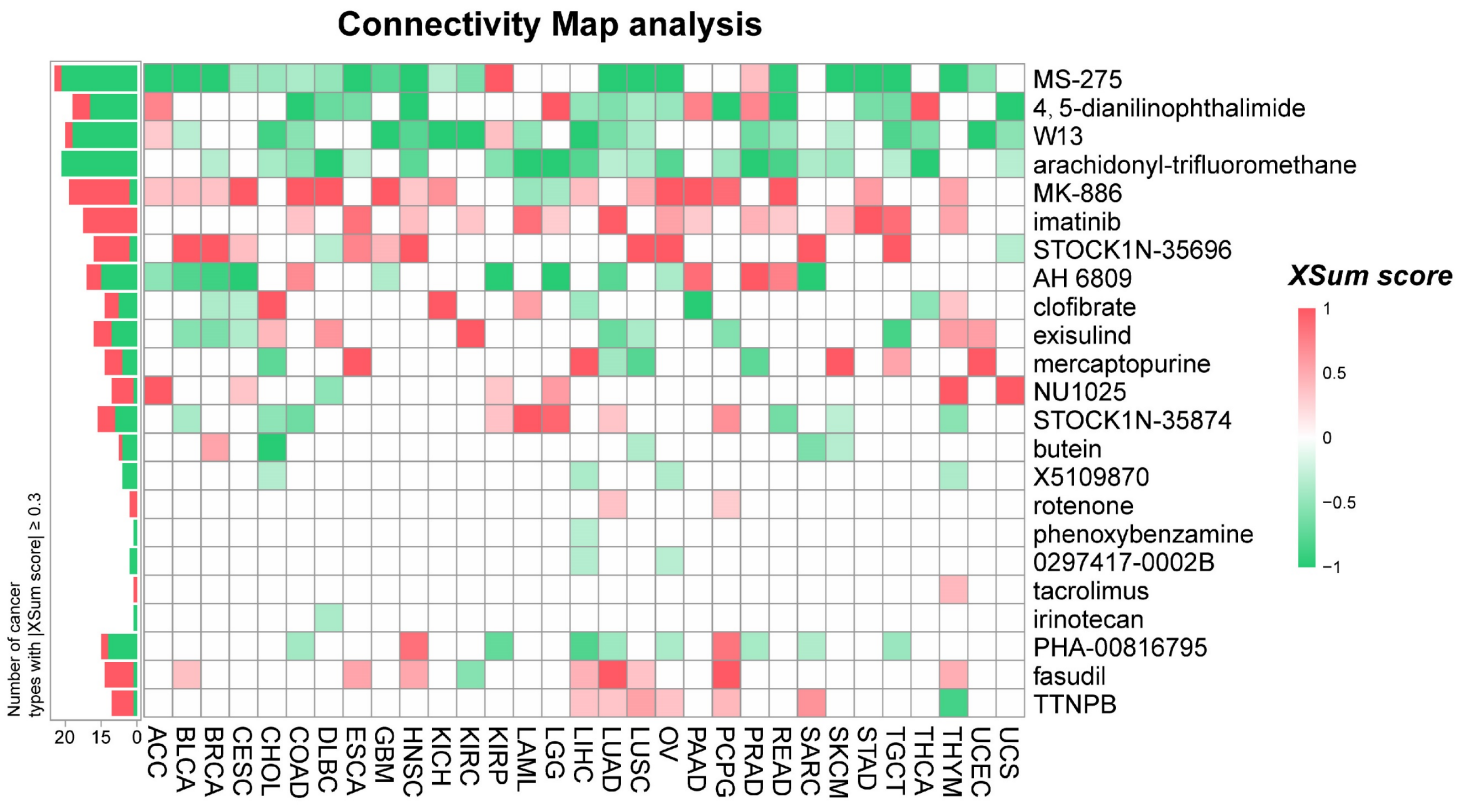
** Prediction of potential compounds targeting NUSAP1.** A heatmap presentation shows the 23 candidate compounds with |XSum| ≥ 0.3 that may target NUSAP1 based on the connectivity map analysis in 31 cancer types. The color codes from red to green represent the XSum score from high (1) to low (-1), respectively.

To explore potential therapeutic options that can counteract the tumor-promoting effects mediated by NUSAP1, we conducted CMap analysis. We constructed a NUSAP1-related gene signature comprising the 500 most significantly upregulated and 500 most significantly down-regulated genes, identified through a comparison of *NUSAP1*-high and *NUSAP1-*low expressing patients in each cancer type. Using the eXtreme Sum (XSum), an optimal signature matching method, we compared the NUSAP1-related signature with CMap gene signatures, resulting in similarity scores for 1288 compounds. Heatmap clustering analysis showed the enrichment of 23 compounds with |Xsum score| ≥ 0.3 across the 31 cancer types. Notably, four compounds, namely MS-275 (Entinostat), 4,5-dianilinophthalimide (DAPH), W-13, and arachidonyl-trifluoromethane (AACOCF3), exhibited significantly lower scores in most cancer types, indicating their potential to inhibit pro-oncogenic effects mediated by NUSAP1 (Figure 8 and Supplementary Table S6). Interestingly, previous studies have demonstrated the tumor-suppressive effects of Entinostat and AACOCF3 28, 29. These findings provide substantial support for the validity of our prediction results, although further investigation is warranted to elucidate the underlying mechanism.

### *NUSAP1* knockdown suppresses cell viability in BRCA and LUAD cells

**Figure 9:**
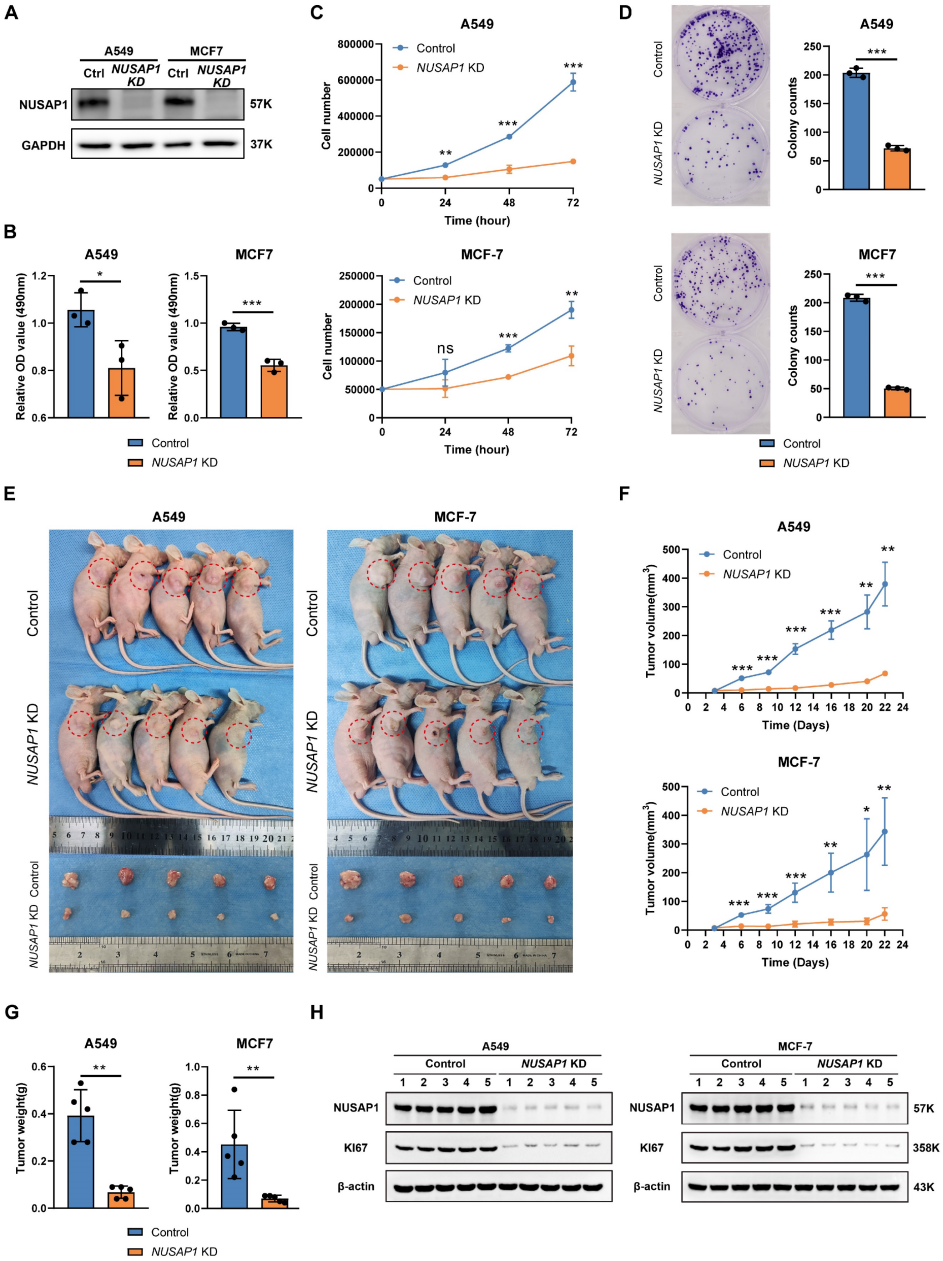
** Knockdown of *NUSAP1* inhibits cell viability and proliferation of A549 and MCF-7. (A)** Knockdown of *NUSAP1* remarkedly reduces the protein levels of NUSAP1 in A549 and MCF-7 cells, respectively. Ctrl, Control.** (B-C)** MTS assay **(B)** and cell count **(C)** demonstrate that *NUSAP1* knockdown inhibits the cell viability of A549 and MCF-7 cells. **(D)** *NUSAP1* knockdown significantly reduces the colony formation ability of A549 (*upper*) and MCF-7 (*lower*) cells. **(E)** Images of control or *NUSAP1* knockdown A549 (*left*) and MCF-7 (*right*) tumor-bearing nude mice and isolated tumors derived from mice. n=5 per group. **(F)** Growth curves of control or *NUSAP1* knockdown A549 (*upper*) and MCF-7 (*lower*) tumors. **(G)** Tumor weight of control or *NUSAP1* knockdown A549 (*left*) and MCF-7 (*right*) tumors. **(H)** Representative images (*left*) of Western blotting showing a decrease in NUSAP1 and KI67 proteins in A549 (*left*) and MCF-7 (*right*) subcutaneous tumors. The histogram shows the intensity of NUSAP1 and KI67 proteins in each subcutaneous tumor. The symbols ns, *, **, and *** indicate not significant, P < 0.05, P < 0.01, P < 0.001, respectively.

To further investigate the functional role of NUSAP1 in tumor cells, we constructed *NUSAP1* stably knockdown cells using A549 (LUAD) and MCF-7 (BRCA) cell lines. The efficiency of *NUSAP1* knockdown was confirmed by Western blot (Figure 9A). MTT assays and cell proliferation experiments demonstrated a significant reduction in cell viability and proliferation upon *NUSAP1* depletion in A549 and MCF-7 cells (Figure 9B-C). The results of colony formation assay also confirmed that *NUSAP1* knockdown greatly decreased the colony formation ability of A549 and MCF-7 cells (Figure 9D).

To further verify the function of NUSAP1 in tumor cell proliferation, we next conducted an *in vivo* experiment. As shown in Figure 9E-9G, the growth rates and tumor weights of A549 and MCF-7 tumors in nude mice were significantly reduced after *NUSAP1* knockdown. The decrease in protein levels of NUSAP1 and KI67, a classic proliferation marker, in A549 and MCF-7 subcutaneous tumors were confirmed by Western blotting (Figure 9H) and IHC results (Supplementary Figure S7). These *in vitro* and* in vivo* results are consistent with the findings from prognostic analyses (Figure 4A) and GESA (Figure 5) reported above. Taken together, our findings highlight the important roles of NUSAP1 in tumorigenesis and cancer progression.

## Discussion

Over the past decade, immunotherapy has proven its ability to prolong the survival of patients with advanced tumors and continues to revolutionize clinical tumor treatment strategies 30. Unfortunately, immunotherapy the response is immunotherapy is limited to only a subset of patients, primarily due to the heterogeneity of tumor-immune microenvironment 31. The identification of factors that can predict the clinical benefit of immunotherapy holds the potential to improve the selection of tumor types and patient subgroups that are likely to respond. In this study, we conducted a comprehensive pan-cancer analysis and uncovered that NUSAP1, an important regulator of mitosis, could serve as a robust predictive biomarker for the prognosis and immunotherapy response of human cancers.

In the expression analysis, we observed high expression of *NUSAP1* in actively proliferating normal testis, whereas its expression was low in relative non-proliferative brain and heart tissues. This expression pattern is likely closely associated with its role in regulating cell mitosis. In addition, single-cell sequencing data of normal tissues revealed that *NUSAP1* was predominantly expressed in epithelial cells. Considering that most solid tumors generally originate from abnormally proliferating epithelial cells, this concentrated expression in epithelial cells suggests that the potential involvement of NUSAP1 in tumorigenesis.

Analysis of public databases and immunohistochemistry (IHC) results confirmed the high expression of NUSAP1 in tumor tissues across various cancer types. This elevated mRNA level was primarily attributed to the upregulation of two protein-encoding transcripts, indicating their potential as targets for *NUSAP1* mRNA interference in tumor. Interestingly, single-cell sequencing data of tumor tissues showed that *NUSAP1* is highly expressed not only in malignant cells but also various immune cells. Notably, NUSAP1 displayed completely opposite expression profiles in proliferating T and exhausted CD8^+^T cells. Recent research revealed the unexpected involvement of the immune cell-activating cytokine interleukin (IL)-2 in inducing T cell exhaustion, establishing a link between cell proliferation and the regulation of immune cell function 32, 33. These findings suggest NUSAP1-mediated cell mitosis might represent a valuable regulatory target for modulating the function of tumor-infiltrating T cells.

In the analysis of prognostic predictive power using OS, DSS, DFI, and PFI, we observed a significant association between *NUSAP1* expression level and prognosis in 26 cancer types. High *NUSAP1* expression was identified as a risk factor in tumorigenesis and progression, especially in four types of kidney cancer (ACC, KICH, KIRC, and KIRP), as well as LGG, LIHC, LUAD, PAAD, PCPG, PRAD, and SARC. However, *NUSAP1* expression showed a positive correlation with a favorable prognosis in CESC, COAD, GBM, HNSC, LUSC, STAD, TGCT, and THYM, indicating a protective role of NUSAP1 in the progression of these cancer types. This finding is also supported by published evidence 8, 10, 11, 14, 34.

Furthermore, we assessed the association between *NUSAP1* expression level and clinical features in these cancer types. Consistent with the survival analysis results, patients with high *NUSAP1* expression in HNSC and STAD exhibited fewer lymph node and distant metastases, as well as lower TNM stage. On the other hand, kidney cancer and LUAD patients with high *NUSAP1* are more prone to metastasis and disease progression. The results from GESA and *in vitro* cell experiments further support this conclusion (Figure 5 and 9B-C). Collectively, these results confirm that *NUSAP1* expression level can serve as a reliable biomarker for predicting the prognosis of patients with various tumors.

It is noteworthy that NUSAP1 appears to play diverse roles in tumor biology, exerting both pro- and anti-tumor effects, despite its high expression in all cancer tissues. The high level of phosphorylation of NUSAP1 in the microtubule binding region inhibits its binding to microtubules, thereby impeding spindle midzone formation and cell cycle progression 23. This potential mechanism may explain the inhibitory effect of NUSAP1 observed in HNSC and other cancer types. Further exploration and elucidation of the molecular mechanism of this difference will be crucial in designing tumor suppressing strategies targeting NUSAP1.

In the analysis of the immunomodulatory function, we found an association between *NUSAP1* expression and immune score of various tumors, indicating that NUSAP1 may have a role in modulating the tumor microenvironment. Further analysis showed that *NUSAP1* expression was positively correlated with the infiltration of Th2, MDSC, and TFH, while showing a negative association with central and effector memory CD4^+^T and NKT cells. Previous reports have demonstrated that Th2 cells exhibit tumor-promoting and immunosuppressive function 24, 35. Furthermore, inhibiting the function and recruitment of MDSC can prevent tumor growth and metastasis 36, and MDSCs have been proved to impair the effectiveness of current anti-tumor strategies such as chemotherapy, radiotherapy, and immunotherapy 37, 38. The reduced infiltration of CD4^+^T and NKT cells, which are key immune cells involved in tumor-killing, in high *NUSAP1* tumors also contributes to an immunosuppressive tumor microenvironment 39-41. In addition, a negative correlation was observed between high *NUSAP1* levels and immune cell infiltration into tumors, as well as the expression of antigen presentation-related molecules in most cancer types. Conversely, a positive correlation was observed between *NUSAP1* expression and various immune negative regulatory molecules. These findings are consistent with the results of GSEA and provide further support for the role of NUSAP1 in mediating immunosuppression within the tumor microenvironment and influencing the efficacy of immunotherapy. Based on the analysis of three cohorts receiving anti-PD-1/PD-L1 therapy, we found that patients with high *NUSAP1* had shorter survival times and lower response rates compared to those with low* NUSAP1*. These results confirm the predictive value of NUSAP1 in determining the response to immunotherapy.

In the CMap analysis, four compounds, Entinostat, DAPH, W-13, and AACOCF3 were identified as potential inhibitor of the pro-oncogenic effects mediated by NUSAP1. Existing evidence suggests that Entinostat and AACOCF3 are inhibitors of histone deacetylases (HDAC) and cytosolic Phospholipase A2 (cPLA2) and have shown high efficacy in inhibiting tumor growth 29, 42, 43. Interestingly, recent research has provided evidence that both treatment with Entinostat or AACOCF3 can promote antitumor immune responses and overcome resistance to PD-1/PD-L1 blockade in breast, lung, and sarcoma tumors 29, 44-46, indicating targeting NUSAP1 may not only inhibit tumor growth but also enhance tumor immunotherapy.

Our previous findings primarily focus on investigating the mechanistic role of NUSAP1 in regulating cell cycle, especially during mitosis. We have demonstrated that NUSAP1 functions as a microtubule spindle stabilizer and an essential regulator of chromosome oscillation during metaphase. NUSAP1 stabilizes mitotic spindle by negatively regulating Mitotic Centromere-Associated Kinesin (MCAK), a microtubule depolymerizer, through its regulation of both the localization and depolymerization activity of MCAK 15. In addition, we have also shown the pivotal role of NUSAP1 in regulating chromosome oscillation by manipulating Kid, a Kinesin Family Member 22, which generates polar ejection force that positively affects chromosome alignment, orientation, and oscillation 16. Depletion of NUSAP1 significantly suppresses the amplitude and velocity of chromosome oscillation, observed as centromere movement during metaphase. Hence, NUSAP1 plays a crucial role in ensuring mitotic fidelity and genetic stability by facilitating the correct formation of mitotic spindle, chromosome alignment, and oscillation. Disruptions in the proper protein level of NUSAP1 can lead to chromosome misalignment and unequal chromosome separation, resulting in genetic instability, a hallmark of cancer. Our current findings further extend our understanding of NUSAP1 from its mechanistic role in regulating genetic integrity to its predictive power for prognosis and immunotherapy response in pan-cancer. This suggests that targeting NUSAP1-mediated microtubule stability could be a novel approach for tumor treatment.

## Conclusions

In conclusion, our study reveals that *NUSAP1* exhibits significant upregulation in most cancer tissues compared to normal tissues, with predominant expression in malignant and immune cells, particularly proliferative T cells. NUSAP1 appears to play a role in promoting cell proliferation and EMT in various cancer types, excluding CESC, HNSC, and STAD. Notably, the expression level of *NUSAP1* holds substantial promise as a valuable biomarker for predicting prognosis and assessing the effectiveness of immunotherapy in human cancers. However, further exploration and verification through additional basic experiments and clinical trials are necessary to elucidate the precise molecular mechanism underlying NUSAP1-mediated functions in tumorigenesis and immunotherapy. These future endeavors will enhance our understanding of NUSAP1's role and its potential as a therapeutic target in cancer treatment.

## Supplementary Material

Supplementary figures.

Supplementary tables.

## Abbreviations

AACOCF3: arachidonyl-trifluoromethane
ACC: Adrenocortical Cancer
ATCC: American Type Culture Collection
BLCA: Bladder Cancer
BRCA: breast cancer
CAF: carcinoma-associated fibroblast
CESC: Cervical Cancer
CHOL: Bile Duct Cancer
COAD: colon cancer
Colorectum: colorectal cancer
CPTAC: Clinical Proteomic Tumor Analysis Consortium
cPLA2: cytosolic Phospholipase A2
DAPH: 4,5-dianilinophthalimide
DC: dendritic cell
DEG: differentially expressed genes
DFI: disease-free interval
DLBC: Large B-cell Lymphoma
DMFS: distant metastases-free survival
DSS: disease-specific survival
EMT: epithelial-mesenchymal transition
Eosi: eosinophils
ESCA: Esophageal Cancer
HNSC: head and neck squamous cell carcinoma
FDR: False Discovery Rate
FP: first progression
GEO: Gene Expression Omnibus
GBM: glioblastoma
GSEA: Gene Set Enrichment Analysis
GTEx: Genotype-Tissue Expression Project
HDAC: histone deacetylases
KICH: Kidney Chromophobe
KIRC: kidney clear cell carcinoma
KIRP: Kidney Papillary Cell Carcinoma
IFN: interferon
IHC: immunohistochemistry
IL: interleukin
LAML: Acute Myeloid Leukemia
LGG: Lower Grade Glioma
LIHC: liver cancer
LUAD: lung adenocarcinoma
LUSC: Lung Squamous Cell Carcinoma
MCAK: Mitotic Centromere-Associated Kinesin
MDSC: myeloid-derived suppressor cell
Mono: monocyte
MSI: microsatellite instability
NES: Normalized Enrichment Score
Neut: neutrophils
NK: natural killer
NUSAP1: Nucleolar and spindle-associated protein 1
NSCLC: non-small cell lung cancer
OS: overall survival
OV: ovarian cancer
PAAD: pancreatic cancer
PCPG: Pheochromocytoma & Paraganglioma
PRAD: prostate cancer
PFI: progression-free interval
PFS: progression-free survival
PPS: post-progression survival
READ: Rectal Cancer
RFS: recurrence-free survival
SARC: Sarcoma
STAD: stomach cancer
SKCM: melanoma
TCGA: the Cancer Genome Atlas
TFH: follicular helper T cell
TGCT: Testicular Cancer
THCA: Thyroid Cancer
THYM: Thymoma
TMB: tumor mutation burden
TME: tumor immune microenvironment
TIP: Tracking Tumor Immunophenotype
TISCH: Tumor Immune Single-cell Hub
Treg: regulatory T cell
UCEC: Endometrioid Cancer
UCS: Uterine Carcinosarcoma
xSum: eXtreme Sum
