# Supplementary material for: Comprehensive pan-cancer analysis reveals NUSAP1 is a novel predictive biomarker for prognosis and immunotherapy response
Source: Int J Biol Sci. 2023 Sep 4;19(14):4689–708. doi: 10.7150/ijbs.80017 (PMC10535697; doi:10.7150/ijbs.80017)
Supplement: Supplementary file 1 — Supplementary figures. [file ijbsv19p4689s1.pdf]

Supplementary Material

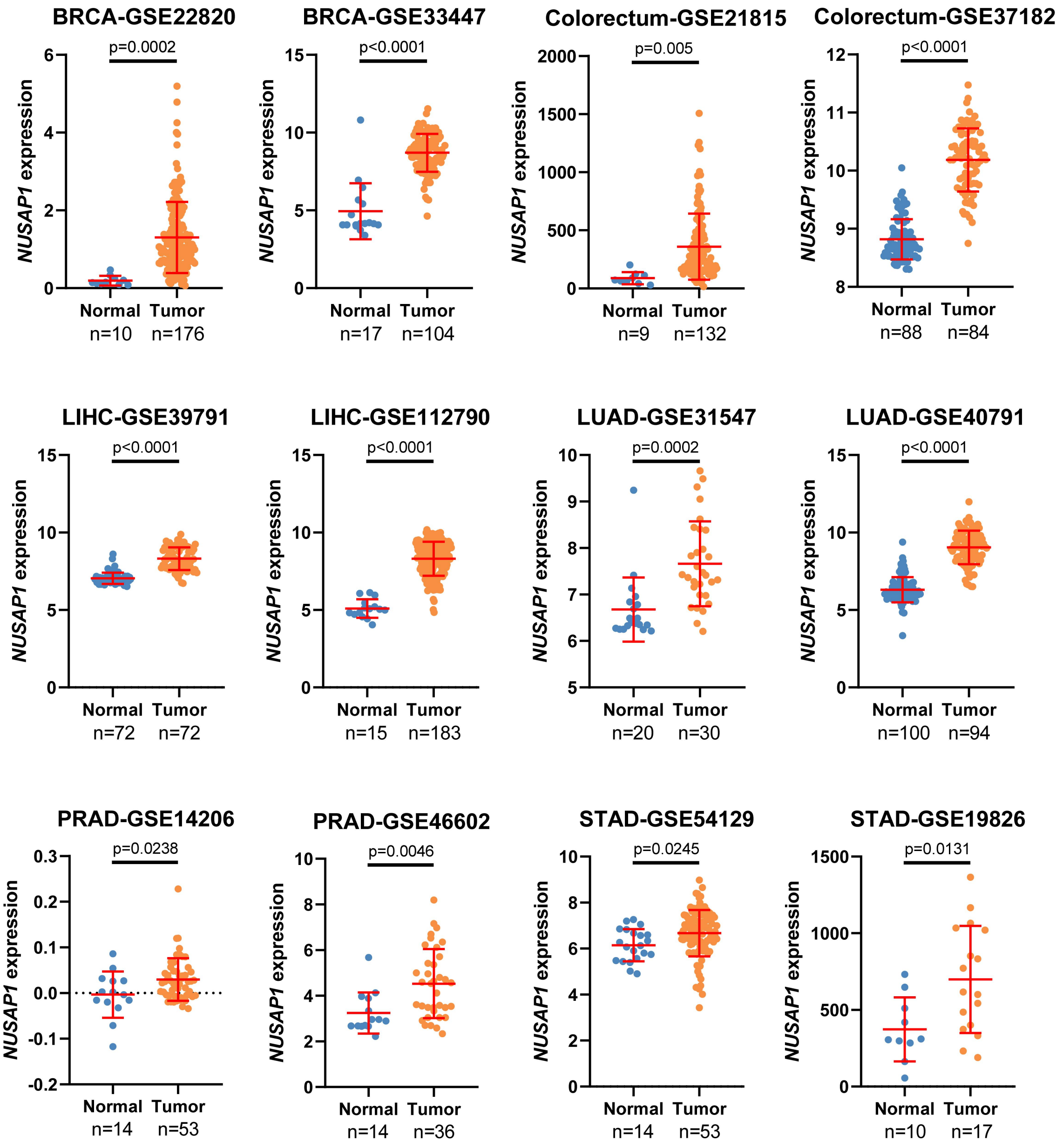

Supplementary Figure S1. Expression of NUSAP1 in GEO database. Comparison of *NUSAP1* expression level between normal and tumor tissues of breast cancer (BRCA), colorectum, liver cancer (LIHC), lung adenocarcinoma (LUAD), prostate cancer (PRAD), and stomach cancer (STAD) in GEO datasets. Blue dots represent each normal sample, and yellow dots represent each tumor sample.

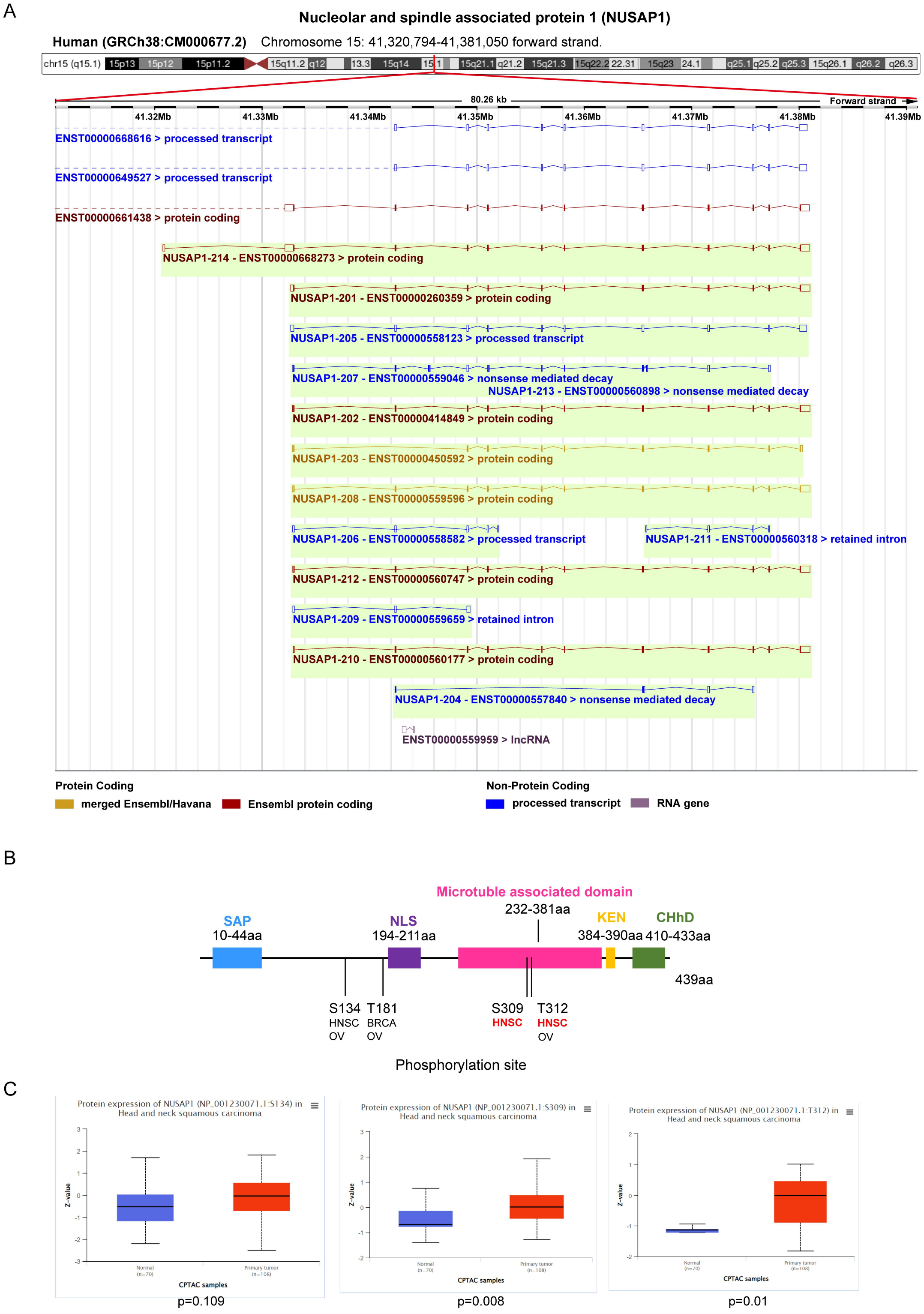

Supplementary Figure S2. Different transcripts and phosphorylation site of human NUSAP1. (A) Location of 13 different transcripts of human NUSAP1 gene in Chromosome 15. **(B)** Functional domain structure of human NUSAP1 protein. **(C)** Comparison of NUSAP1 protein level with different phosphorylation sites in HNSC tumor and normal tissues using CPTAC database. Blue box represents normal tissue and red box represents tumor tissue.

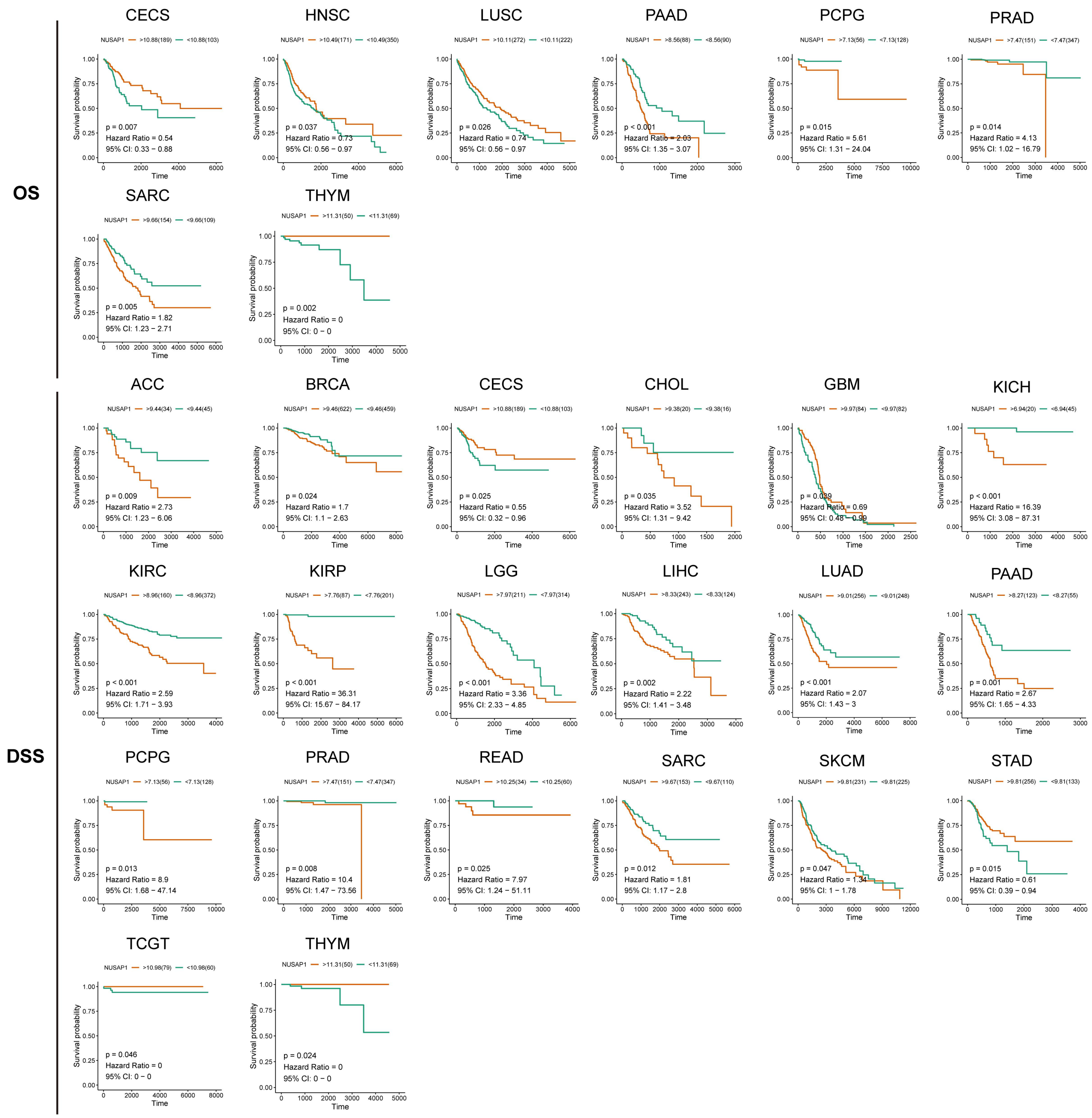

Supplementary Figure S3. Predictive value of *NUSAP1* expression in overall survival (OS) and disease-free survival (DSS) in pan-cancer. Survival analysis was performed using curated survival data from TCGA database and R package (survival, v3.3-1; survminer, v0.4.9).

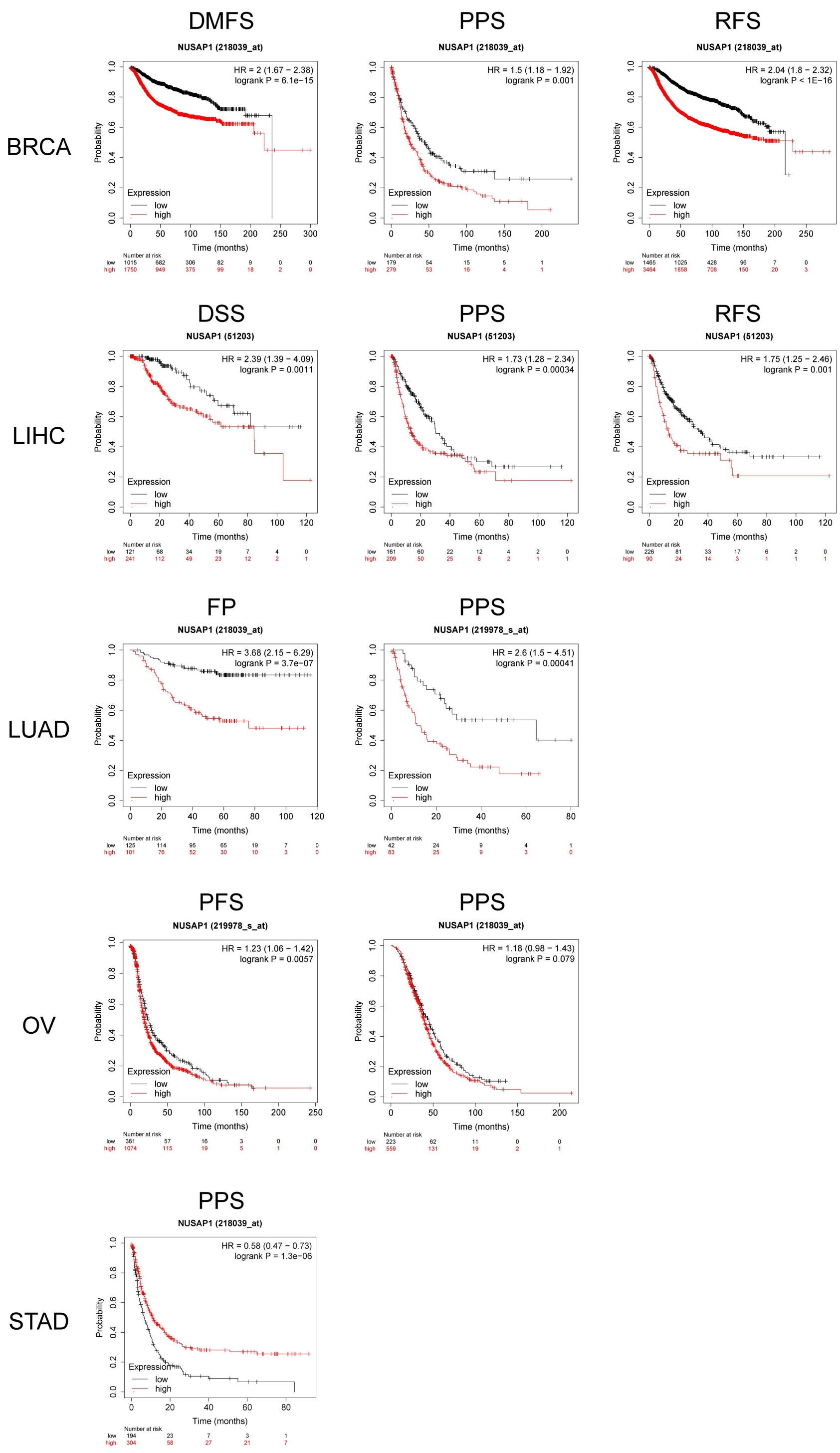

Supplementary Figure S4. Predictive value of *NUSAP1* expression in (distant metastasis-free survival) DMFS, Post-Progression Survival (PPS), progression-free survival (PFS), relapse-free survival (RFS), DSS, first progression (FP) in BRCA, LIHC, LUAD, OV and STAD. Survival analysis was performed using data from GEO databases and KM plot online tool.

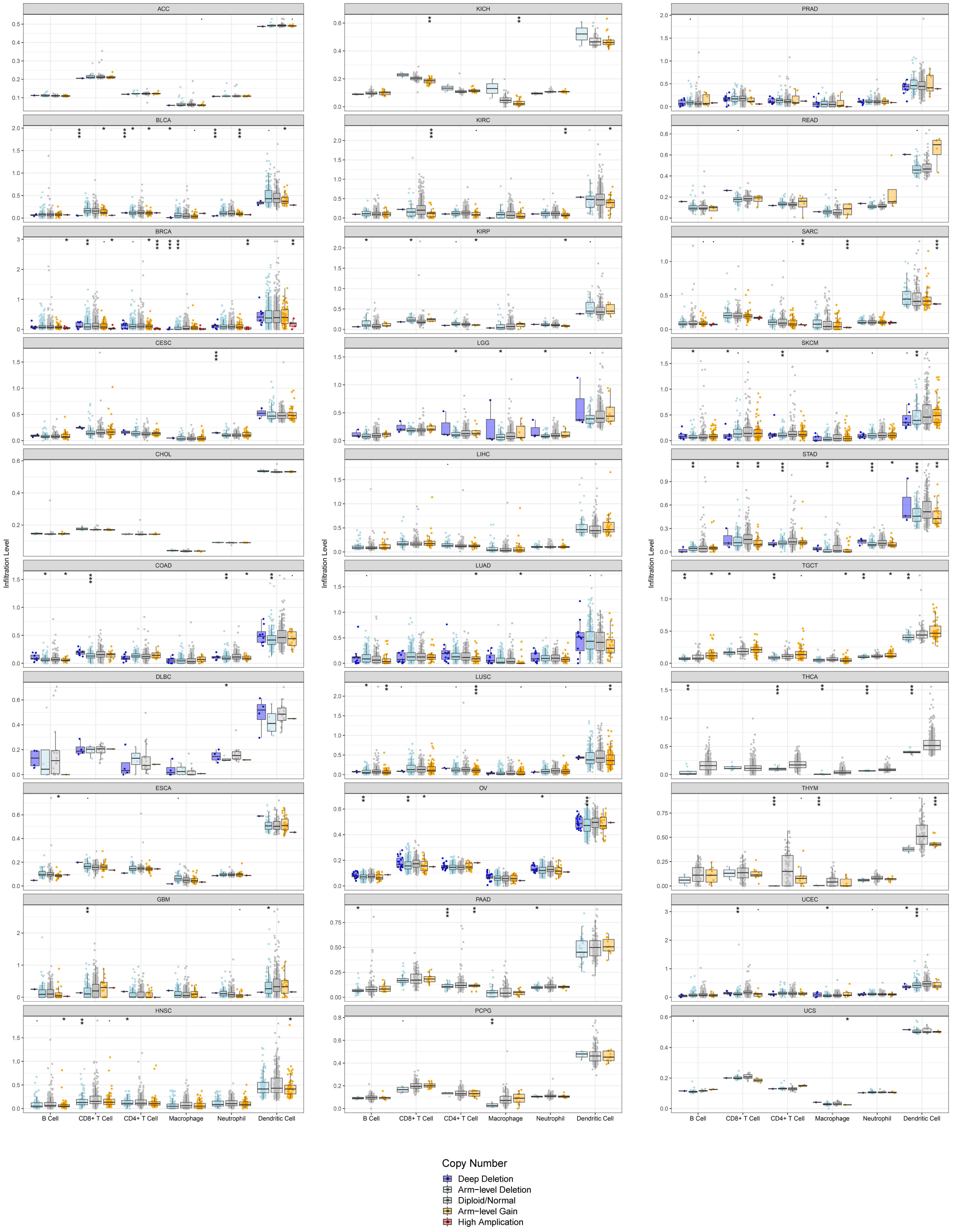
ACC
KICH
PRAD
BLCA
KIRC
READ
BRCA
KIRP
SARC
CESC
LGG
SKCM
CHOL
LIHC
STAD
COAD
LUAD
TGCT
DLBC
LUSC
THCA
ESCA
OV
THYM
GBM
PAAD
UCEC
HNSC
PCPG
UCS
Infiltration Level
B Cell
CD8+ T Cell
CD4+ T Cell
Macrophage
Neutrophil
Dendritic Cell
Copy Number
Deep Deletion
Arm−level Deletion
Diploid/Normal
Arm−level Gain
High Amplication

Supplementary Figure S5. Infiltration of six immune cell types in tumor patients with different *NUSAP1* mutation status. Box plots are generated for each immune subset, to compare the distributions of B, $CD4^{+}T$, $CD8^{+}T$, Macrophage, Neutrophil, and Dendritic cells infiltration levels under different mutation status of *NUSAP1*, with statistical significance estimated using two-sided Wilcoxon rank-sum test.

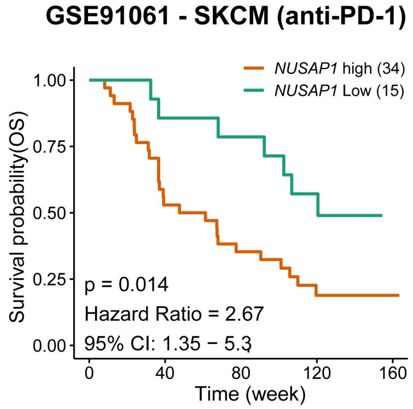

Supplementary Figure S6. Predictive values of *NUSAP1* expression on OS of SKCM patients in anti-PD-1/PD-L1 immunotherapy. KM survival analysis was performed using data from GEO databases (GSE91061) to show the difference of OS between NUSAP1-high and -low melanoma patients after PD-1/PD-L1 treatment.

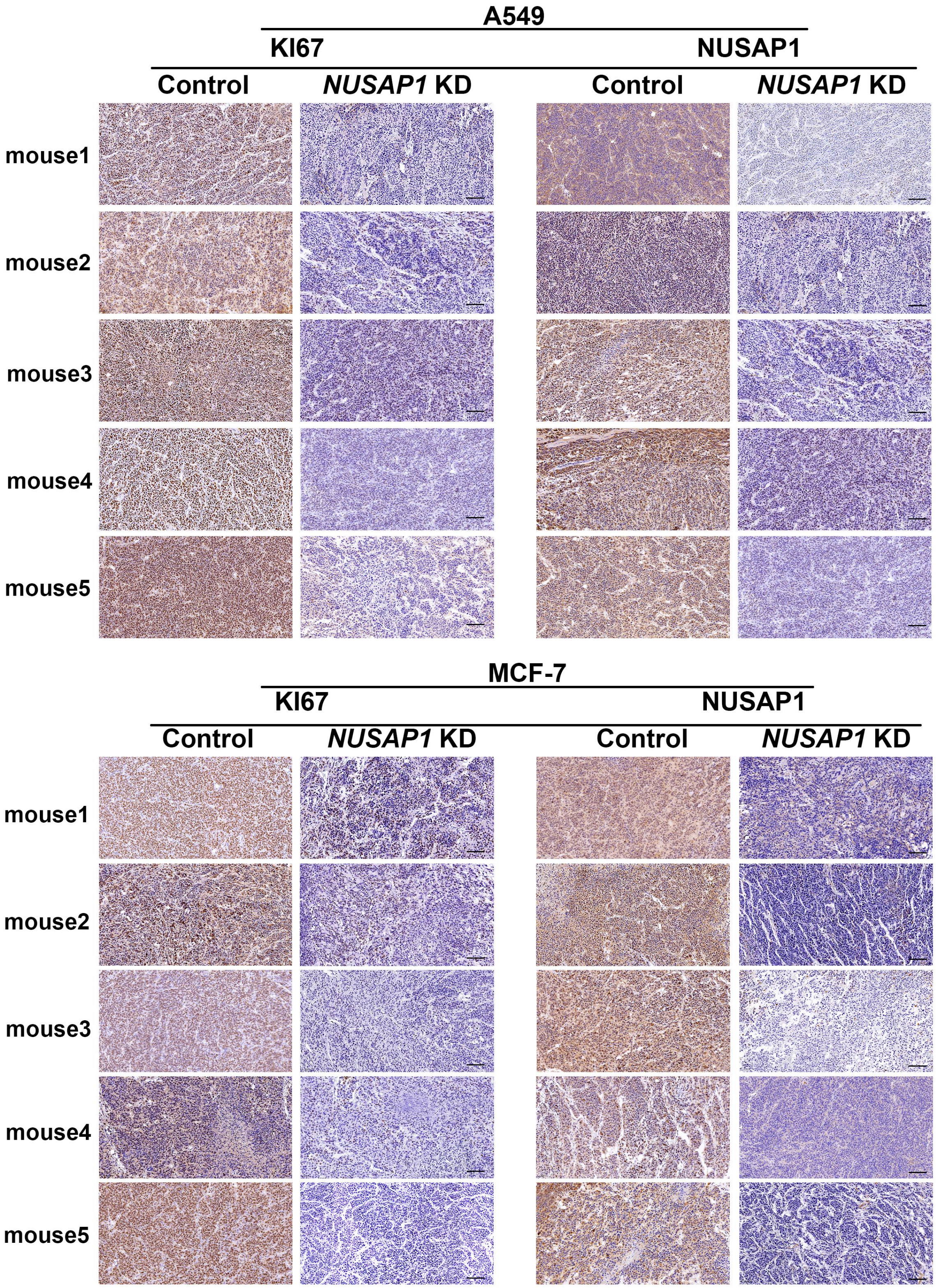

Supplementary Figure S7. Results of histochemical staining of KI67 and NUSAP1 proteins in A549 (*up*) and MCF-7 (*down*) subcutaneous tumor tissues from control and *NUSAP1* knockdown (*NUSAP1* KD) groups. Scale bar =100 μm.
